# Genomic analyses with biofilter 2.0: knowledge driven filtering, annotation, and model development

**DOI:** 10.1186/1756-0381-6-25

**Published:** 2013-12-30

**Authors:** Sarah A Pendergrass, Alex Frase, John Wallace, Daniel Wolfe, Neerja Katiyar, Carrie Moore, Marylyn D Ritchie

**Affiliations:** 1 Center for Systems Genomics, Department of Biochemistry and Molecular Biology, The Pennsylvania State University, Eberly College of Science, The Huck Institutes of the Life Sciences, University Park, Pennsylvania, PA, USA

**Keywords:** Data mining, Bioinformatics, Expert knowledge, Modeling, Pathway analyses, Epistasis

## Abstract

**Background:**

The ever-growing wealth of biological information available through multiple comprehensive database repositories can be leveraged for advanced analysis of data. We have now extensively revised and updated the multi-purpose software tool Biofilter that allows researchers to annotate and/or filter data as well as generate gene-gene interaction models based on existing biological knowledge. Biofilter now has the Library of Knowledge Integration (LOKI), for accessing and integrating existing comprehensive database information, including more flexibility for how ambiguity of gene identifiers are handled. We have also updated the way importance scores for interaction models are generated. In addition, Biofilter 2.0 now works with a range of types and formats of data, including single nucleotide polymorphism (SNP) identifiers, rare variant identifiers, base pair positions, gene symbols, genetic regions, and copy number variant (CNV) location information.

**Results:**

Biofilter provides a convenient single interface for accessing multiple publicly available human genetic data sources that have been compiled in the supporting database of LOKI. Information within LOKI includes genomic locations of SNPs and genes, as well as known relationships among genes and proteins such as interaction pairs, pathways and ontological categories.

Via Biofilter 2.0 researchers can:

• ***Annotate*** genomic location or region based data, such as results from association studies, or CNV analyses, with relevant biological knowledge for deeper interpretation

• ***Filter*** genomic location or region based data on biological criteria, such as filtering a series SNPs to retain only SNPs present in specific genes within specific pathways of interest

• ***Generate Predictive Models*** for gene-gene, SNP-SNP, or CNV-CNV interactions based on biological information, with priority for models to be tested based on biological relevance, thus narrowing the search space and reducing multiple hypothesis-testing.

**Conclusions:**

Biofilter is a software tool that provides a flexible way to use the ever-expanding expert biological knowledge that exists to direct filtering, annotation, and complex predictive model development for elucidating the etiology of complex phenotypic outcomes.

## Background

Expanding resources of existing knowledge can be used to direct new analyses investigating the relationship between genetic architecture and outcome traits, as well as provide more information for interpreting analysis results. Many different types of “-omic” analyses, such as genome-wide association studies (GWAS) or eQTL analyses, take a high-throughput association approach with the multiple hypothesis testing burden and potential for Type-1 error increasing with increasing numbers of SNPs and/or phenotypes/outcomes tested for association in these studies. Existing expert-knowledge can be used to filter data in various ways before calculating associations, thus reducing the number of proposed tests and the multiple testing burden based on a biologically-driven rationale. Existing biological data can also be used to annotate results of high-throughput studies to provide further biological interpretation of genomic regions showing statistically significant associations.

Further, methodologies that facilitate the exploration of models beyond those explored through the GWA approach are important for potentially explaining more of the heritability of complex traits. The approach of comprehensive single-variant associations with outcome(s) do not explore gene-by-gene (G×G), SNP-by-SNP (SNP×SNP), or higher order n-way models. Models that consider epistasis, or the interaction between genetic variants, may explain the some of the “missing-heritability” of complex traits [1,2]. Unfortunately, investigating interactions with comprehensive association testing can result in much higher multiple hypothesis testing and increased Type-1 error, due to the number of n-wise combinations to explore. The calculation of all possible n-way combinations may even become computationally intractable when the number of combinations is high enough. Thus there is a key need for high-throughput strategies for prioritizing model testing, particularly when expanding to higher order genetic models.

We designed the software tool Biofilter [3] to allow researchers to leverage biological knowledge existing across multiple databases to annotate genomic information, inform choices of SNPs for association testing, as well to provide a biologically driven way for developing more complex GxG models. Biofilter has been used successfully for biologically driven knowledge gene-gene interaction analyses for several diverse outcomes: HDL-Cholesterol levels [4], virologic failure with efavirenz-containing HIV treatment regimens [5], multiple-sclerosis [6], and cataracts [7].

The new version of Biofilter described herein (version 2.0) shares some features of previous versions of Biofilter, however the software has been almost entirely re-engineered. Improvements include the ability to work with an increased range of genomic data types and formats of data, including SNP identification numbers (rs numbers), base pair locations, copy number variants (CNV), gene ID, and/or genomic region location information. The diverse biological knowledge distilled from multiple external databases has been expanded, updated, and integrated into a flexible single database called the Library of Knowledge Integration (LOKI) that we will continue to expand with additional database sources over time. The software now also provides multiple options for handling the ambiguity of gene/protein identifiers that can exist within the various data sources, allowing the user to choose the level of ambiguity which is acceptable on a case-by-case basis when generating pairwise models. The data within LOKI is complex, thus the new software now comes with a separate, un-changing, *simulated knowledge database* that can be used for evaluating Biofilter features and clarifying how various settings may modify Biofilter output.

Biofilter can be used to generate biological-information derived pairwise G×G, SNP×SNP, or CNV-by-CNV (CNV×CNV) based interaction models. Biofilter has access to thousands of biological connections and groupings between genes and proteins, thus can identify pairs of genes (and in extension pairs of SNPs or CNVs) appearing together over the widest array of original data sources. Each pair-wise model generated in Biofilter has an “implication-index” based on the degree of repeated patterns for genes within the prior knowledge database. With increasing implication-index there is more potential for biologically important interactions between SNPs or genes or CNVs.

Pair-wise models identified with Biofilter can then be tested for statistical significance with any number of association methods (Biofilter is not specific to any particular statistical method). Through choosing a model implication score cutoff the number of models to test can be reduced, thus avoiding the prohibitive computational and multiple-testing burden of an exhaustive pairwise analysis. Even while the number of models to test is reduced, because of the nature of Biofilter, there is a biological foundation supporting the relevance of statistically significant results.

Biofilter can also be used to annotate data or results with relevant biological knowledge for data analysis and interpretation. Biofilter also allows for filtering data based on biological criteria, allowing researchers to harness information from multiple sources in a number of potential ways for the reduction of data for analysis and/or interpretation.

Advanced tools for genomic analyses are critical for effectively using the wealth of genomic data and published research available. Biofilter is one of these advanced tools, and using Biofilter may help to elucidate a new picture of the relationship between genetic architecture and phenotypic outcomes such as the presence or absence of disease through facilitating a directed exploration of higher-order interaction models. Further, the features of Biofilter allow researchers to gain more information about –omic data through using the wealth of existing and ever-growing biological information existing in the public domain.

### Implementation

Biofilter was developed in Python, and functions on the command line for Linux, Mac OS X or Windows based machines. Additional file 1, the Biofilter 2.0 manual, is provided here as a resource with extensive details beyond the scope of this manuscript, as well as examples of commands and configuration file formats.

### Running biofilter

Biofilter can be run from a command-line terminal by executing “biofilter.py” (or “python biofilter.py”) and specifying the desired inputs, outputs and other optional settings. All options can either be provided directly on the command line (such as “biofilter.py --option-name”) or placed in one or more configuration files whose filenames are then provided on the command line (such as “biofilter.py analysis.config”). The former approach may be more convenient for setting up the necessary options to achieve the desired analysis, but the latter approach is recommended for any final runs, since the configuration file then serves as a record of the Biofilter analysis. Any number of configuration files may be used, with options from later files overriding those from earlier files, and options on the command line override those from any configuration file.

### Data types

**Figure 1:**
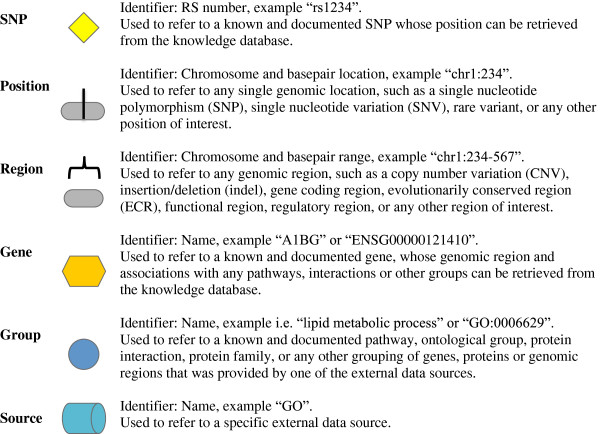
Data types of Biofilter: Biofilter can work with and understand the relationships between six basic types of data presented in table, along with shapes representing each data type.

Biofilter can work with and identify the relationships between six basic data types explained in detail in Figure 1: SNP, Position, Region, Gene, Group, and Source data. Single base-pair data to be used within Biofilter can be referred to via RS number or chromosome and base-pair position. Due to the change of RS numbers over time as consensus is reached for various SNPs, using chromosome and base-pair number is the most reliable loci identification to use. Gene, region, and CNV data can also be used within Biofilter. Again, using a chromosome and base-pair range can be a more reliable source of region data, as gene identifiers can sometimes refer to more than one region in Entrez.

Biofilter uses the most recent Genome Reference Consortium build at the time of installation (use command --report-genome-build/REPORT_GENOME_BUILD to determine the current reference build being used by Biofilter). Thus, software like LiftOver may need to be used if a different reference assembly is the initial starting data, in order to convert loci information from one assembly to another (http://genome.ucsc.edu/cgi-bin/hgLiftOver).

*Sources* and *groups* are how data within LOKI are designated. *Sources* are the external sources that are downloaded and integrated into LOKI, such as Gene Ontology (GO). Each known and documented pathway, ontological group, protein interaction, protein family, or any other grouping of genes, proteins or genomic regions provided by one of the external data sources is considered a *group*.

### Library of Knowledge Integration (LOKI)

**Figure 2:**
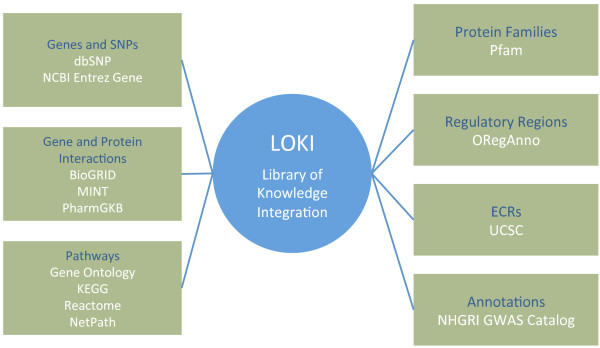
The Library of Knowledge Integration (LOKI) contains information from multiple database repositories.

Rather than issuing queries in real-time to a series of external databases, Biofilter consults a local database called LOKI, visualized in Figure 2. This local repository contains all the knowledge from downloaded raw data from each external source. The current databases compiled in LOKI are presented in Table 1.

**Table 1:** Knowledge Sources within the Library of Knowledge Integration (LOKI)

**Source**	**URL**	**Citation**	**Summary**
**BioGRID**	http://thebiogrid.org	[8]	BioGRID is a repository with genetic and protein interaction data from model organisms and humans used by Biofilter for linking position and region data to interaction information.
**NCBI dbSNP**	http://www.ncbi.nlm.nih.gov/snp		A database of SNPs and multiple small-scale variations including insertions, deletions, microsatellites and non-polymorphic variants. This resource includes a complete list of known human SNPs and their base pair positions relative to the human reference genome. Biofilter uses the data of dbSNP in two ways: connecting SNP identifiers (RS numbers) of dbSNP to genomic positions and connecting retired identifiers to current identifiers.
**NCBI gene**	http://www.ncbi.nlm.nih.gov/gene		Entrez is a search engine that allows researchers to search many discrete health sciences databases at the NCBI. The database provides an extensive list of known human genes, their beginning and ending base pair positions, and many alternate names and cross-referenced database identifiers. This data is used in two ways within Biofilter: to connect gene symbols to their genomic regions, and to connect equivalent gene symbols and identifiers to each other.
**Gene ontology**	http://www.geneontology.org	[9]	The Gene Ontology database defines terms representing gene product properties, such as cellular components, molecular function, and biological processes, within a hierarchical tree of ontology groups and related proteins.
**MINT**	http://mint.bio.uniroma2.it/mint/Welcome.do	[10]	The Molecular Interaction database contains experimentally verified protein-protein interactions from the scientific literature, which are used in Biofilter for linking position and region data to interacting protein pairs.
**NetPath**	http://www.netpath.org	[11]	The NetPath database consists of curated human signaling pathways which are used by Biofilter.
**OregAnno**	http://www.oreganno.org/oregano	[12]	The Open REGulatory ANNOtation database is used by Biofilter for curation information about known regulatory elements from the scientific literature.
**Pfam**	http://pfam.sanger.ac.uk	[13]	The Pfam database is a large collection of protein families. The annotation of data respective to proteins within Biofilter is based on the information from Pfam.
**PharmGKB**	http://www.pharmgkb.org	[14]	Biofilture currently uses this database for pathway based data, future releases of Biofilter will also include drug-related data of gene-drug associations and pharmacological association study results.
**Reactome**	http://www.reactome.org/ReactomeGWT/entrypoint.html	[15]	Biofilter uses the information contained in Reactome to establish pathway and network relationships between genes.
**UCSC genome browser**	http://genome.ucsc.edu	[16]	This source provides access to a growing database of genomic sequence and annotations for a wide variety of organisms, currently we use the UCSC for location information for evolutionary conserved regions (ECRs) for Biofilter and to acess OregAnno’s regulatory region data.
**NHGRI GWAS catalog**	http://www.genome.gov/gwastudies/	[17]	A catalog of published GWAS SNP-trait associations with p-values < 1.0 × 10–5, for studies with at least 100,000 SNPs assayed

LOKI must be generated on the local system before Biofilter can be used. Because the resulting knowledge database is a single local file, Biofilter itself does not require a network connection to run and can be run locally. Biofilter is released with a loader script that will automatically query the database sources listed in Table 1 to compile into the local LOKI database. LOKI requires 10–20 GB of temporary storage during the process of the automatic building of the database, and requires 5–10 GB for the final knowledge database file. Further specific details for LOKI installation and updating LOKI are available within Additional file 1.

It is important to note that the various data sources integrated into LOKI can be updated at any time. This new data will not be available to Biofilter until the LOKI knowledge database is updated or regenerated. The Biofilter software release includes a script so that end-users can update their local LOKI database as desired. We recommend that researchers become familiar with how often data sources are updated and plan to update LOKI accordingly, preferably at least once every few months.

If a given set of analyses needs to be repeatable or verifiable, such as those published in a manuscript, we recommend storing an archived version of the LOKI knowledge database from the time of the analyses. These archived versions of the database can then be used to repeat or augment an analysis based on exactly the same prior knowledge, regardless of any updates that may have occurred in various data sources afterwards. The LOKI build script offers a “finalize” function which can help facilitate database archiving by reducing the file size and also preventing any future attempts to update the same database file. It may also be helpful to include the date in the filename of each newly compiled version of LOKI in order to carefully distinguish between older versions.

### LOKI simulation for biofilter exploration

**Figure 3:**
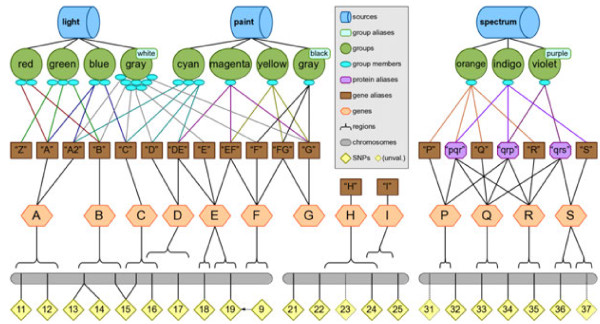
**The *simulated knowledge database *for exploration of Biofilter functionality.** Biofilter has a simulated knowledge database so that researchers can try features of the software and see the results, with the ability to track what happened in the process. The above schematic shows that the database contains three fictitious database sources: “light”, “paint” and “spectrum”. The sources are connected to eleven groups via lines: “red”, “green”, “blue”, “gray”, “cyan”, “magenta”, “yellow”, “gray”, “orange”, “indigo”, and “violet”. These sources/groups contain group members (blue ovals) that are linked to gene aliases (brown boxes). These gene aliases then link to 13 genes (orange hexagons), and three chromosomes (grey bars) with 21 SNPs (yellow diamonds). The gene aliases provide examples of ambiguous gene identifiers. Groups cannot presently be connected to more than one source, however groups may have more than one name provided by the source that defines them. For example, in the real source of KEGG, each KEGG pathway (group) has both a numeric ID number as well as a textual pathway name. In the schematic, the two nodes labeled “gray” are intended to depict two separate groups (provided by two separate sources) which happen to share the “gray” identifier, but which each also have another name which is distinct (“white” and “black”). Additional file 1 contains example commands used with this simulated database, along with the resultant output.

We have built an accessible, limited, and unchanging *simulated knowledge database* for exploring filtering, annotation, and model building commands for Biofilter 2.0, visualized in Figure 3. This database contains three fictitious sources named “light”, “paint” and “spectrum”. These sources are linked to eleven groups named “red”, “green”, “blue”, “gray”, “cyan”, “magenta”, “yellow”, “gray”, “orange”, “indigo” and “violet”. These groups collectively have three chromosomes, 13 genes, and 21 SNPs. The manual for Biofilter 2.0, Additional file 1, contains wide range of example commands used with Biofilter and this simulated knowledge base, along with the resultant output. This simulated knowledge base is released with Biofilter as a means for researchers to perform testing or validation of commands.

## Results and discussion

### Biofilter: overview

**Figure 4:**
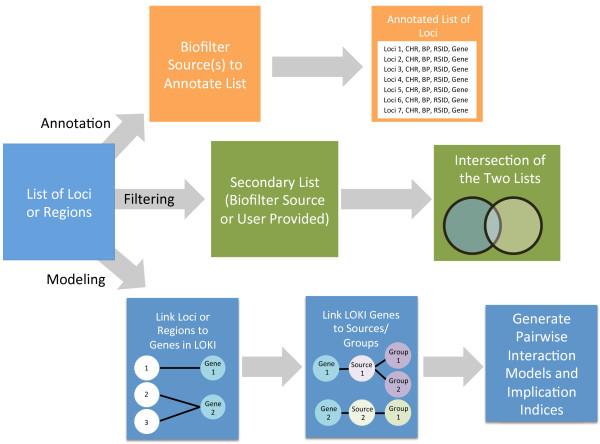
**Basic Overview of Biofilter. *Annotation:*** Biofilter allows researchers to annotate a list of loci with information from LOKI. For example, if a list of chromosome and base pair locations are provided, the researcher might want annotation with the list of current SNP identifiers for those loci, as well as what gene the SNP might be located within. ***Filtering:*** Biofilter allows a researcher to filter an input list (such as a list of SNPs) by another list (a list of genes), and then Biofilter will provide a list of those SNPs that only fall within the list of genes. ***Modeling*** starts with an input list of loci or regions that are then linked to gene regions in LOKI, those genes are then linked out to sources and groups. As a final step pairwise interaction models with implication indices are generated.

As mentioned, Biofilter has three primary analysis modes which each make use of the available biological knowledge in slightly different ways: Filtering, Annotation, and Modeling. For the purpose of annotation and filtering, Biofilter takes a list of loci or a list of regions, and then either filters that list by another list (whether provided as input or using information from LOKI), or annotates the list provided. For the development of models based on existing biological knowledge, the input is a list of loci (whether SNPs or chromosome and base pair locations), or a list of regions (such as genes), and these are first mapped to known protein-coding gene regions by Biofilter. In the case of CNVs, Biofilter by default maps CNVs to genes by considering CNVs with even one base-pair of overlap as mapped to a given gene. The researcher can change the degree of overlap required for CNVs to map to genes according to preference. Connections are then automatically forged between this resultant list of genes, and any instances of these genes within the sources of LOKI. As a result, starting from a list of loci or regions, Biofilter connects that list to additional existing information. Figure 4 provides a diagram of how this filtering, annotating, and modeling with Biofilter works.

We provide here further details and examples of filtering, annotating, and modeling within Biofilter, although it is important to note that annotation, filtering, and modeling with Biofilter are not exclusive, and can be combined to analyse data according to a researchers preferences.

### Filtering

**Figure 5:**
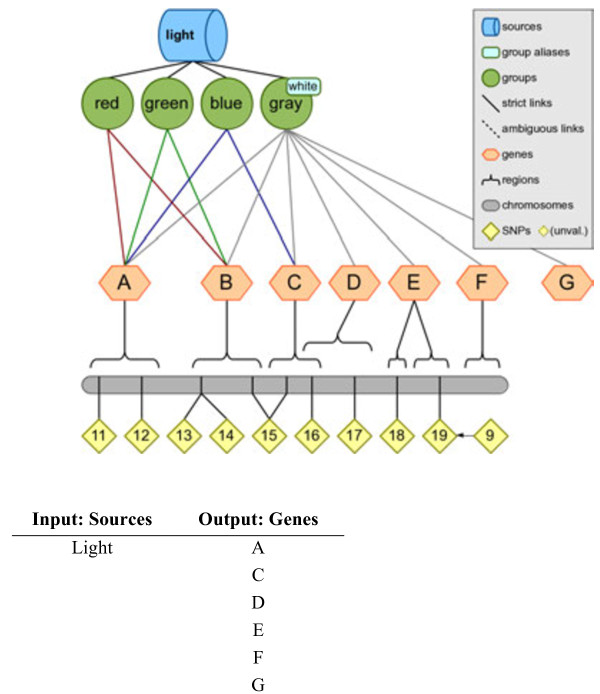
**Filtering Example: Output a list of all genes within a data source.** This figure shows an example of using Biofilter obtain a list of all genes within a given source. The input of “light” was used with the simulated knowledge database, and the result was a list of all “genes” within the source.

**Figure 6:**
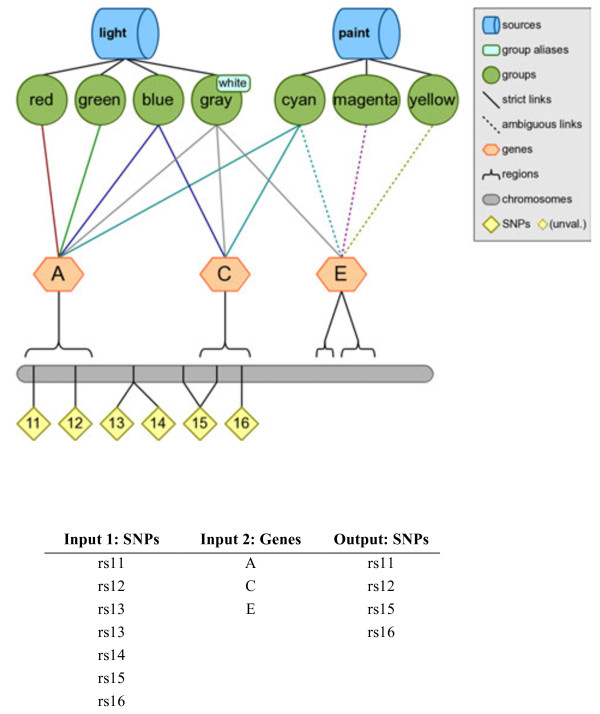
**Filtering example: Filtering a list of SNPs by a list of genes, using the simulated data set.** This figure shows an example of filtering using Biofilter to obtain, out of a specific list of SNPs, only those SNPs within a specific set of genes. The input to Biofilter is one list of SNPs *Input 1* and one list of genes *Input 2*. These lists correspond to the “SNPs” and “genes” of the simulated knowledge base. If Input 1 is filtered by Input 2, the result is four SNPs.

The most straightforward of Biofilter’s primary functions is, as the name implies, filtering. Given any combination of input data, Biofilter can cross-reference the input data using the relationships stored in the knowledge database to generate a filtered dataset of any supported type (or types). For example, a very straightforward use of Biofilter would be to obtain the list of all genes within a specific data source in LOKI, as visualized using the simulated knowledge database in Figure 5. Another example of filtering with Biofilter would be to use a list of SNPs (such as those covered by a genotyping platform) and a list of genes (such as those thought to be related to a particular phenotype) and then using Biofilter to request the set of SNPs existing within those genes. Biofilter will use LOKI’s knowledge of SNP positions and gene regions to filter the provided SNP list, removing all those that are not located within any of the provided genes. Figure 6 shows an example of this using the simulated knowledge database, and the resultant filtered output.

The output data type does not necessarily have to be the same data type(s) provided as input. For example, a researcher can provide a list of SNPs and a list of groups and request the set of genes that match both lists. In this case, there is no input set of genes to use as a starting point so Biofilter will check all known genes found in the knowledge database. The result is a list of only the genes which include at least one of the specified SNPs, and are a part of at least one of the specified groups.

Finally, filtering is not limited to a single data type: Biofilter can also identify all of the unique combinations of data types which jointly meet the provided criteria. For example, given a list of SNPs and genes, Biofilter can produce a filtered set of SNP-gene pairs. The result is every combination of SNP and gene from the two lists where the SNP is within the gene, or within a user-defined window around the gene.

### Annotation

Biofilter can also annotate any of the supported data types with respect to any of the others. Like filtering, the annotations are based on the relationships stored in the knowledge database; unlike filtering, any data which cannot be annotated as requested (such as a SNP which is not located within any gene) will still be included in the output, with the annotation columns of the output simply left blank.

**Figure 7:**
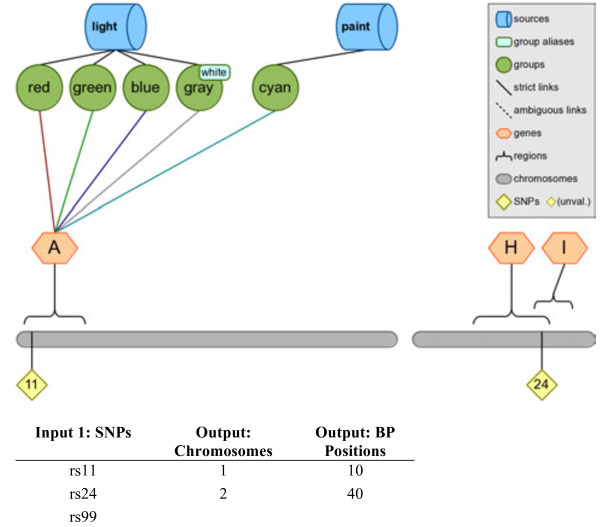
**Example of annotation, obtaining more information for specific SNPs.** This figure shows an example of filtering using Biofilter annotate SNPs with gene information. The output in this case is matched by row to the input. The SNP “rs99” does not exist within the simulated knowledge database, so the SNP has no annotation. These SNPs could be further annotated with gene symbol and gene-region annotation. Note that SNP “rs24” is in within genes “H” and “I”, thus if gene annotation was also added here the output the SNP would listed twice, with each row showing a different gene identifier.

For example, a list of SNPs can be annotated with positions to generate a new list of the SNPs with extra columns containing the chromosome and genomic position for each SNP (if any), we show an example in Figure 7. Any SNP with multiple known positions will be repeated, and any SNP with no known position will have blanks in the added columns.

**Figure 8:**
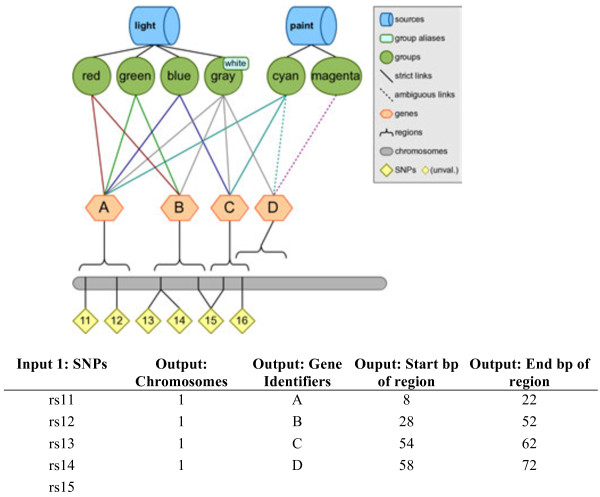
**Example of annotation, obtaining more information about genes SNPs are within.** This figure shows an example of providing a list of SNPs and then receiving as output the list of genes the SNPs are within. Information about the start base pair and end base pair of the genes are also listed in the output.

Similarly, those same SNPs can be annotated with gene information; the result is similar, except that the added column contains the name of the gene containing the SNP’s position. In this case a blank value can mean two things: either the SNP does not fall within any known gene region, or the SNP has no known position with which to search for gene regions. Figure 8 shows an example of this kind of annotation, using the simulated knowledge database. For another example, a researcher could also annotate a list of gene symbols with SNPs, regions, groups, and sources, using Biofilter.

Annotations can also be generated for combinations of data types, or for data types which were not provided as input. In these cases the annotation will be for the output of a filtering analysis. For example, a researcher could provide a list of SNPs and a list of groups, and then request an annotation of genes to regions. Since no genes were provided as input, Biofilter will first identify all genes which contain at least one of the provided SNPs, that are also part of at least one of the provided groups. This filtered set of genes will then appear in the first column of the annotation output, followed by each gene’s genomic region (if any).

As an example use for region based data, such as copy-number variation data, base pair start-and-stop regions can be provided to Biofilter, and then that data can be annotated with gene information using Biofilter, based on percent of overlap or number of base-pairs overlapped.

Filtering can also be followed by annotation. For example, Biofilter can be used to find the overlapping SNPs between the two lists and then map the overlapping SNPs to genes, regions, groups and the sources.

### Modeling

The last of Biofilter’s primary analysis modes is a little different from filtering and annotation. In addition to simply cross-referencing any given data with the other available prior knowledge, Biofilter can also search for repeated patterns within the prior knowledge that might indicate the potential for important interactions between SNPs or genes.

Any pathway, ontological category, protein family, experimental interaction, or other grouping of genes or proteins represents a relationship between those genes or proteins. Two genes appearing together in more than one grouping are likely to have an important biological relationship, and two genes appearing in multiple groups from several independent sources are even more likely to be biologically related in some way.

Biofilter modeling is “gene-focused”, and can take any combination of input data, map that data to genes, then search LOKI for likely pairwise interaction models. Thus, a list of SNPs can be developed and gene-gene models can be requested from Biofilter; Biofilter will then only consider models in which the genes contain at least one of the specified SNPs. For another example, if SNPxSNP models are requested, Biofilter will take each baseline gene-gene model, separately map the two genes to all applicable SNPs, and then return all possible pairings between those two sets of SNPs.

The resultant models suggested by Biofilter are ranked in order of likelihood, using an “implication index.” This score is simply a combination of two tallies: the number of original data sources which contained the pair, and the number of different groups among those sources. For example a score of “2-3” indicates that the model appears in three different groups, and those groups originated with two different sources.

**Figure 9:**
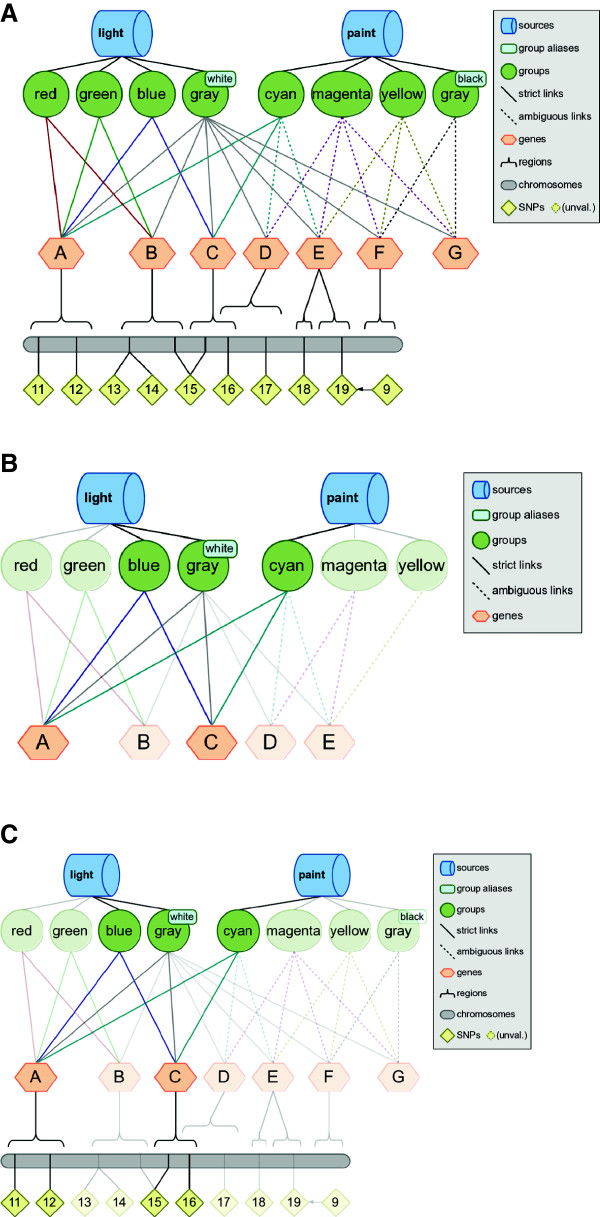
**Modeling Example using Biofilter.** This is an example of mapping the input list of SNPs to genes within Biofilter; using all of the SNPs on the first “chromosome” (the grey bar at the bottom of **(A)**). Note that Gene F does not contain any SNPs. Biofilter will then connect, pairwise, the genes that contain pairs of SNPs from the input list of SNPs. Genes A and C are found together in three groups across two sources **(B)**. The other genes on the first chromosome were not found as a pair in any of the other groups. Both the light and paint sources contain groups—blue, gray, and cyan—that suggest a relationship between genes A and C. This relationship will be summarized by the implication score “2-3,” which gives the number of sources followed by the number of groups which support this gene model. Each time the same pairwise model of genes is found in another source, the left-hand index of the implication score for that pairwise model increases by one; each time it is found in another group from the same source, the right-hand index increases by one. Biofilter will next break down the gene-gene models into all pairwise combinations of SNPs across the genes within sources light and paint **(C)**, resulting in pairwise combinations of the SNPs rs11, rs12, rs15, and rs16.

For an example, perhaps a researcher has provided a list of SNPs, all of the SNPs on the first “chromosome” of the simulated knowledge base in Figure 3. These SNPs are found within two sources and eight pathways shown in Figure 3. The researcher would like to generate pairwise SNP-SNP models using Biofilter. So, after supplying the list of SNPs, Biofilter will first map the input list of SNPs to genes within Biofilter. Note in Figure 3 that Gene F does not contain any SNPs, so Gene F will not be included in the resultant Gene-Gene models, shown in Figure 9. Next the genes that contain SNPs in the input list of SNPs will be connected pairwise. Biofilter will determine that genes A and C are found together in three groups across two sources, the *light* and *paint* sources contain groups—*blue, gray*, and *cyan*—that suggest a relationship between genes A and C, as seen in Figure 9. Thus, this relationship is summarized by the implication score “2-3,” which gives the number of sources followed by the number of groups which support this gene model. Each time the same pairwise model of genes is found in another source, the left-hand index of the implication score for that pairwise model increases by one; each time it is found in another group from the same source, the right-hand index increases by one. In the last step, the gene-gene models are broken down into all pairwise combinations of SNPs across the genes within sources *light* and *paint*, as seen in Figure 9. Biofilter 2.0 will automatically generate gene models prior to generating SNP models and there is no need to specify any of these steps separately.

### Using resultant models

A researcher can choose an implication score cutoff of choice, balancing the number of associations to perform with the implication support of models of interest. Then the researcher can use their statistical approach of choice for investigating the significance of the interaction models.

### Ambiguity and biofilter

One of the changes to Biofilter 2.0 is handling ambiguity for genes or groups. Any given gene or group might go by many different names in different contexts, and the new version of Biofilter can accommodate this ambiguity depending on researcher preference. For example, there are names associated with more than one gene; these names are considered ambiguous. For example, although *A1B* is an alias of the gene *A1BG*, it is also an alias of the gene *SNTB1* (syntrophin, beta 1). Therefore if *A1B* appears in an input gene list file, Biofilter will not inherently recognize which gene was intended for inclusion (*A1BG* or *SNTB1*).

It is important to note that SNP annotations to genes will not change from source to source, SNP identifiers will either map to genes (depending on the gene boundaries set by the user), or SNPs will not map to genes. The user is provided with feedback indicating SNPs, input to Biofilter, that are not mapped to genes.

When an ambiguous gene or group identifier appears in an input file, Biofilter has two options: include all genes or groups with which the identifier is associated, or none of them. A warning is displayed in either case, and options are also available to generate a detailed report of the ambiguous identifiers.. Thus, for the *A1B* example, the researcher can decide if they will map *A1B* to *A1BG* and *SNTB1*, and keep both genes in further analyses, or drop both out of further analyses, through choice of the option ALLOW_AMBIGUOUS_GENES. Ambiguous group names are only important if the user wishes to provide an input list of groups in order to limit their analysis. If the user provides an ambiguous group name, Biofilter’s behavior is similar to the case of ambiguous gene names: a warning will be displayed, and Biofilter will either include all groups which match the name or none of them, according to the option ALLOW_AMBIGUOUS_GROUPS.

For the gene identifier data within the prior knowledge sources of LOKI however, the situation can become more complicated because many sources provide more than one identifier for each member of a group. For example in a KEGG pathway definition, each gene that makes up the pathway is specified both by its Entrez Gene ID number and by its symbolic abbreviation. If either of the pair of identifiers are connected to more than one gene, or the pair of identifiers are connected to different genes, then it is impossible for LOKI to know with certainty which gene is supposed to be part of the group.

Rather than attempting to compromise on a “one size fits all” approach to this ambiguity, Biofilter supports several different options for interpreting ambiguity. Each of these interpretations comes with a slightly different trade-off between false-positives and false-negatives, and the number of resultant models. The ambiguity interpretation most appropriate to the task can be selected by the researcher at run-time, as Biofilter’s results can change depending on the choice for handling ambiguity.

The most conservative approach is to simply disregard any data which is ambiguous. This ensures that Biofilter will not report any false-positive annotations or models, but true annotations may be missing from the output as a result. This “strict” interpretation is the only one that was supported in earlier versions of Biofilter, and it remains the default mode in Biofilter 2.0.

At the opposite extreme, when there is any doubt about which gene belongs in a group, Biofilter can proceed as if every candidate is a member of the group. This “permissive” approach ensures that no true annotation will be missing from the output, but it will also cause false annotations to be reported.

Between these two extremes, Biofilter also supports two different heuristic strategies for reducing ambiguity. These strategies essentially make an educated guess about what the original data source intended by the set of identifiers it provided. The first heuristic is called “*implication*” and it rates the likelihood of each potential gene being the intended one by counting the number of identifiers which implicate that gene. The second heuristic, called “*quality*,” is similar, but considers the number of genes that each identifier refers to as a measure of that identifier’s quality; a high-quality identifier (which refers to only one or two genes) is then given more weight than a low-quality identifier (which refers to many genes).

In practice, these two heuristic strategies will often produce the same results; in fact, when using real data from our real prior knowledge sources, we have yet to find a case where they do not reach the same conclusion. It is possible that such a case will arise in the future, however, so we have incorporated these two heuristics into Biofilter 2.0.

The researcher can indicate which heuristics, if any, should be employed to mitigate ambiguity in the prior knowledge database. The permissible values for this option are “implication” or “quality” to employ a specific heuristic strategy, or “no” or “any”. When set to “no”, no attempt is made to reduce ambiguity and all genes implicated by any of the provided identifiers are considered equally likely interpretations. When set to “any” then all heuristics are attempted simultaneously and the winner(s) from each one are added to the group; if both heuristics chose only a single winner but they disagree with each other, then both would be added, although this has never been observed in practice.

A researcher can choose, in addition to which heuristics they want to use (if any), either a “strict” or “permissive” option. When using the strict option, none of the possible genes will be considered a member of the group if there are multiple possibilities. When enabled with the permissive option, the most-likely possibilities will *all* be included.

It should also be noted that if the user chooses a heuristic or permissive approach instead of the “strict” default, then some extra (possibly incorrect) annotations or models may be reported as a result of ambiguity, and these will not be differentiated from the other results. If there is any question about the consequences of using ambiguous data, the results can always be compared to the same analysis run in “strict” mode.

**Figure 10:**
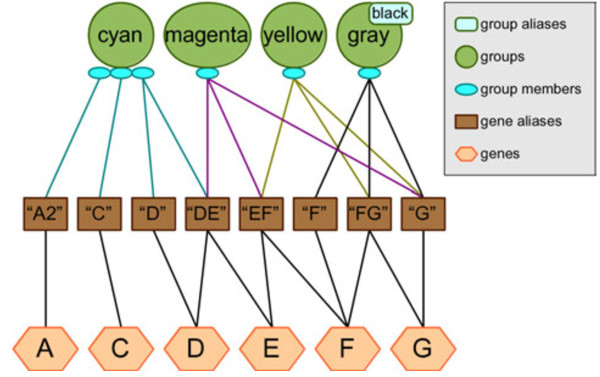
**Ambiguity example within the Biofilter simulated knowledge database.** The testing knowledge included with Biofilter contains several examples of these kinds of situations, depicted in the diagram. Note that this diagram reflects the fact that there may be multiple names for the same gene (i.e. “D” and “DE” both refer to gene D), and some names may be associated with multiple genes (i.e. “DE” refers to both genes D and E). The “cyan” group contains three genes, of which the third is ambiguous because we are given two identifiers for it, but one of them refers to two different genes. The “magenta”, “yellow” and “gray/black” groups each contain only one gene, but in each case we are given three different names for that. Because of the ambiguity in the provided identifiers, genes considered members of these groups will appear to vary depending on the researcher’s choice for ambiguity settings.

We show in Figure 10 and example of ambiguity that is incorporated into the simulated knowledge database, allowing researchers to explore the way output changes when using different ambiguity settings. The simulated knowledge database included with Biofilter contains several examples of potential ambiguity situations, depicted in detail in Additional file 1.

### Protein identifiers and ambiguity

So far, our depiction of ambiguity in the knowledge database has implied that groups always contain genes. This allows for the convenient assumption that when we are given more than one identifier for something in a group, we are expecting all of those identifiers to refer to one (and only one) gene.

The reality is, of course, a little more complicated: some sources provide groups that actually contain proteins. In order to make this knowledge compatible with the rest of the prior knowledge, LOKI must translate these protein references into genes, but this breaks that convenient assumption. If a group contains genes then we can reasonably expect each member of the group to be a single gene, but if the group contains proteins, then we must be prepared for a single protein-member to correspond to many genes.

To account for this, LOKI differentiates between identifiers which refer directly to genes (such as symbolic abbreviations or Entrez Gene ID numbers) and identifiers which refer to proteins (such as UniProt ID numbers) that may in turn correspond to many genes.

If any of the identifiers provided for one member of a group is a protein identifier, LOKI disregards any non-protein identifiers. If there is only one protein identifier, then LOKI considers all genes which correspond to that protein to be members of the group, with no ambiguity. If there are multiple protein identifiers then there may be ambiguity if they do not correspond to the same set of genes.

Since protein identifiers are expected to correspond to multiple genes, the concept of an identifier’s “quality” no longer has meaning; consequently, whenever protein identifiers are involved, the implication and quality heuristic strategies become functionally equivalent. In both cases, a gene’s likelihood of being associated with a group is proportional to the number of protein identifiers which implicated it. When no heuristics are used, then all genes which are implicated by any of the protein identifiers are considered equally likely to belong in the group. The simulated knowledge database included with Biofilter also contains several examples of groups with protein identifiers.

## Conclusions

Herein we have presented an updated version of Biofilter 2.0. We have detailed the new Library of Knowledge Integration (LOKI) that provides the database of integrated publicly available biological knowledge that can easily be updated to keep pace with the ever growing data repositories. To handle ambiguity we have designed multiple approaches, to allow researchers to customize the handling of ambiguity within data sources dependent on preference. We have also developed a simulated knowledge database for exploring options and commands with Biofilter.

Biofilter can be used with a range of different data types. As long as the data are position (single base pair) or region (base pair – base pair) based data, they can be used with Biofilter. Biofilter can thus be used with variant but also CNV or other genomic data. Biofilter is also flexible in terms of the analyses that can be performed. We have described here examples of using Biofilter for filtering, annotation, and modeling, with examples focused specifically on SNPs and genes. Another example use of Biofilter is filtering variants based on biological criteria, to reduce the number of variants to be used in eQTL studies, allowing for the number of association tests to be used by filtering variants based on specific pathways or groups of interest.

Our future directions include design of a graphical interface for Biofilter. We also will be adding additional data sources to LOKI, as new databases of biological annotation and expert knowledge are being introduced at a steady pace. We will be developing functionality for n-wise GxG and SNPxSNP model development, allowing biological knowledge to be used for data reduction for models beyond pairwise interactions. We will also be exploring permutation testing approaches for evaluating implication-score significance. Information about non-protein coding regions is increasing, and we also intend to expand the functionality of biofilter for non-protein coding and regulatory regions of the genome. The use of Biofilter now, and moving forward, will allow researchers more tools for exploring the association between genetic variation and outcome, by leveraging the wealth of knowledge collected through research.

### Availability and requirements

#### Prerequisites

The following prerequisites are required to compile the LOKI database and run Biofilter:

• Python, version 2.7 or later

• Python module “apsw” (Another Python SQLite Wrapper)

• SQLite, version 3.6 or later

Note that the dependency on SQLite may be satisfied via the “apsw” Python module, since it often comes with an embedded copy of the necessary SQLite functionality.

The LOKI prior knowledge database must be generated before Biofilter can be used. This is done with the “loki-build.py” script that is installed along with Biofilter. There are several options for this utility, detailed in Additional file 1.

**Project name:** Biofilter 2.0

**Project home page:**http://www.ritchielab.psu.edu

**Operating systems(s):** Linux, Mac OS X, Windows

**Programming language:** Python, SQLite

**License:** GNU General Public License

**Any restrictions to use by non-academics:** The use of Biofilter 2.0 is restricted to academic and non-profit researchers

## Competing interests

The authors declare that they have no competing interests.

## Authors’ contributions

AF and JW programmed; SAP, AF, JW, MDR, DW, NK, CM participated in design of the software; SAP, AF, MDR, DW, participated in the drafting of the manuscript. All authors read and approve the final manuscript.

## Supplementary Material

Additional file 1 The detailed manual for Biofilter 2.0. Click here for file
