# Supplementary material for: Genomic analyses with biofilter 2.0: knowledge driven filtering, annotation, and model development
Source: BioData Min. 2013 Dec 30;6:25. doi: 10.1186/1756-0381-6-25 (PMC3917600; doi:10.1186/1756-0381-6-25)
Supplement: Additional file 1 — The detailed manual for Biofilter 2.0. [file 1756-0381-6-25-S1.pdf]

Biofilter
User's Guide

version 2.1

The information contained in this document is the sole property of the lab of Dr. Marylyn Ritchie
Unauthorized reproduction is prohibited. Last updated July 18, 2013

Table of Contents

Introduction 6
What is Biofilter? 6
Why use Biofilter? 6
Library of Knowledge Integration (LOKI) 6
Knowledge Sources 7
Data Types 8
Analysis Modes 9
Filtering *9*
Annotation *9*
Modeling *10*
Primary and Alternate Input Datasets 10
Identifiers 11

Installation & Setup 12
Prerequisites 12
Platforms 12
Installing Biofilter 12
Compiling Prior Knowledge 12
LOKI Build Script Options *13*
Updating & Archiving Prior Knowledge 14
LD Profiles 15

Using Biofilter 16
Configuration Options 16
--help / HELP *16*
--version / VERSION *16*
--report-configuration / REPORT_CONFIGURATION *16*
--report-replication-fingerprint / REPORT_REPLICATION_FINGERPRINT *17*
Prior Knowledge Options 17
--knowledge / KNOWLEDGE *17*
--report-genome-build / REPORT_GENOME_BUILD *17*
--report-gene-name-stats / REPORT_GENE_NAME_STATS *17*
--report-group-name-stats / REPORT_GROUP_NAME_STATS *17*
--allow-unvalidated-snp-positions / ALLOW_UNVALIDATED_SNP_POSITIONS *17*
--allow-ambiguous-knowledge / ALLOW_AMBIGUOUS_KNOWLEDGE *17*
--reduce-ambiguous-knowledge / REDUCE_AMBIGUOUS_KNOWLEDGE *17*
--report-ld-profiles / REPORT_LD_PROFILES *18*
--ld-profile / LD_PROFILE *18*
--verify-biofilter-version / VERIFY_BIOFILTER_VERSION *18*
--verify-loki-version / VERIFY_LOKI_VERSION *18*
--verify-source-loader / VERIFY_SOURCE_LOADER *18*
--verify-source-option / VERIFY_SOURCE_OPTION *18*
--verify-source-file / VERIFY_SOURCE_FILE *18*
Primary Input Data Options 18
--snp / SNP *18*
--snp-file / SNP_FILE *19*
--position / POSITION *19*
--position-file / POSITION_FILE *19*
--region / REGION *19*
--region-file / REGION_FILE *19*
--gene / GENE *19*

--gene-file / GENE_FILE ... 19
--gene-identifier-type / GENE_IDENTIFIER_TYPE ... 19
--allow-ambiguous-genes / ALLOW_AMBIGUOUS_GENES ... 20
--gene-search / GENE_SEARCH ... 20
--group / GROUP ... 20
--group-file / GROUP_FILE ... 20
--group-identifier-type / GROUP_IDENTIFIER_TYPE ... 20
--allow-ambiguous-groups / ALLOW_AMBIGUOUS_GROUPS ... 20
--group-search / GROUP_SEARCH ... 20
--source / SOURCE ... 20
--source-file / SOURCE_FILE ... 20
Alternate Input Data Options ... 21
--alt-snp / ALT_SNP ... 21
--alt-snp-file / ALT_SNP_FILE ... 21
--alt-position / ALT_POSITION ... 21
--alt-position-file / ALT_POSITION_FILE ... 21
--alt-region / ALT_REGION ... 21
--alt-region-file / ALT_REGION_FILE ... 21
--alt-gene / ALT_GENE ... 21
--alt-gene-file / ALT_GENE_FILE ... 21
--alt-gene-search / ALT_GENE_SEARCH ... 22
--alt-group / ALT_GROUP ... 22
--alt-group-file / ALT_GROUP_FILE ... 22
--alt-group-search / ALT_GROUP_SEARCH ... 22
--alt-source / ALT_SOURCE ... 22
--alt-source-file / ALT_SOURCE_FILE ... 22
Positional Matching Options ... 22
--region-position-margin / REGION_POSITION_MARGIN ... 22
--region-match-percent / REGION_MATCH_PERCENT ... 22
--region-match-bases / REGION_MATCH_BASES ... 23
Model Building Options ... 23
--maximum-model-count / MAXIMUM_MODEL_COUNT ... 23
--alternate-model-filtering / ALTERNATE_MODEL_FILTERING ... 23
--all-pairwise-models / ALL_PAIRWISE_MODELS ... 23
--maximum-model-group-size / MAXIMUM_MODEL_GROUP_SIZE ... 23
--minimum-model-score / MINIMUM_MODEL_SCORE ... 23
--sort-models / SORT_MODELS ... 23
Output Options ... 24
--quiet / QUIET ... 24
--verbose / VERBOSE ... 24
--prefix / PREFIX ... 24
--overwrite / OVERWRITE ... 24
--stdout / STDOUT ... 24
--report-invalid-input / REPORT_INVALID_INPUT ... 24
--filter / FILTER ... 24
--annotate / ANNOTATE ... 24
--model / MODEL ... 24

Input File Formats ... 25
Configuration Files ... 25
SNP List Input Files ... 26
Position Data Input Files ... 26
Region Data Input Files ... 27

Gene and Group List Input Files 27
Source List Input Files 28

Output File Formats 29
Configuration Report 29
Gene and Group Name Statistics Reports 29
LD Profiles Report 30
Invalid Input Reports 30
Analysis Outputs 30

Example Knowledge 33

Example Commands 35
Filtering Examples 35
Example 1: Filtering a list of SNPs by a genotyping platform, where input1 is the first list of SNPs and input2 is the list of SNPs on the genotyping platform. 35
Example 2: Output a list of SNPs from a genotyping platform that correspond to a list of genes. 36
Example 3: Input a list of groups, output regions within those groups. 37
Example 4: Output a list of all genes within a data source. 37
Example 5: Start with a list of genes, output all the genes within particular groups. 38
Example 6: Start with genes associated with a pathway or group, output genes within that group that overlap with an input list of genes. 39
Example 7: Starting with a list of genes, determine genes are within a group. 39
Annotation Examples 39
Example 1: Annotating a SNP with gene region information. 39
Example 2: Annotating SNPs with location information. 40
Example 3: Map a SNP to the groups and sources where the SNP is present. 41
Example 4: Annotating a base pair region with the list of SNPs in that region. 41
Example Filtering followed by annotation 42
Example 1: Input a SNP list and map SNP positions to regions. 42
Example 2: Map SNPs to groups and filter on the source. 42
Example 3: Testing overlap of SNP and region lists, outputting regions. 43
Example 4: Testing overlap of gene and source lists, outputting regions. 43
Example 5: Filter gene list based on sources, and output regions. 44
Example 6: Output of genes found in pathway based input, filtered by genotyping platform. 44
Example 7: Output of genes annotated by group found in pathway based input, filtered by genotyping platform. 45
Example 8: Genes within data sources from a list of input genes filtered by genotyping platform, output regions. 45
Example 9: Find overlap between two SNP lists and map the overlapping SNPs to the genes. 46
Example 10: Find overlapping SNPs between the two lists and map the overlapping SNPs to the genes, regions, groups and the sources. 46
Example 11: Mapping regions to genes using Biofilter based on percent of overlap. 47
Example 12: Mapping regions to genes using Biofilter based on base pair overlap. 48
Example 13: Annotating a list of gene symbols with SNPs, regions, groups, and sources, using Biofilter. 48
Modeling Example 49
Step 1 50
Step 2 50
Step 3 51
Changes in Biofilter 2.0 Modeling 52

Appendix 1: Ambiguity in Prior Knowledge 53
Ambiguity Reduction Heuristics 54
Ambiguity Options 54

Gene Ambiguity Examples 54
Example 1: cyan *54*
Example 2: magenta *55*
Example 3: yellow *55*
Example 4: gray/black *55*
Protein Identifiers 56
Protein Ambiguity Examples 57
Example 1: orange *57*
Example 2: indigo *57*
Example 3: violet *57*

Appendix 2: LD Profiles 58
Installing LD Spline 58
Generating LD Profiles 58
Population Build Script Options *59*

Introduction

What is Biofilter?

Biofilter is a software tool that provides a convenient single interface for accessing multiple publicly available human genetic data sources. These sources include information about the genomic locations of SNPs and genes, as well as relationships among genes and proteins such as interaction pairs, pathways and ontological categories. Biofilter will cross-reference all of this prior biological knowledge in several different ways, with any number of combinations of input data.

Why use Biofilter?

While genome-wide association studies (GWAS) have been used to identify genetic variants that contribute to disease susceptibility on a single-variant single-phenotype level, other approaches can be used to investigate the association between genetic and phenotypic variation. Use of the software tool Biofilter is one such example of a complementary but alternate approach. Biofilter allows users to work with a range of types and formats of data, including SNPs, copy number variant (CNV), and gene location information, along with a repository of diverse biological knowledge distilled from multiple external databases. Via Biofilter, users can annotate data or results with relevant biological knowledge for analysis and interpretation. Biofilter also allows users to filter data based on biological criteria, allowing users to harness information from multiple sources for the reduction of data for analysis. Finally, Biofilter can be used to generate biological-information derived pairwise interaction models for reducing the computational and statistical burden of large-scale interaction data analysis, while also providing a biological foundation to support the relevance of statistically significant results. The use of Biofilter may help to elucidate a new picture of the relationship between genetic architecture and complex phenotypic outcomes such as the presence or absence of disease.

Library of Knowledge Integration (LOKI)

Rather than issuing queries in real-time to a series of external databases, Biofilter consults a local database called the Library of Knowledge Integration, or LOKI. This local repository contains all the knowledge from bulk downloads of the raw data from each external source.

LOKI must be generated on the local system before Biofilter can be used, but because the resulting knowledge database is a single local file, Biofilter itself does not require an internet connection to run. The process of building LOKI requires a relatively large amount of time and disk space to complete, but only needs to be done occasionally to incorporate updated data files from the various sources.

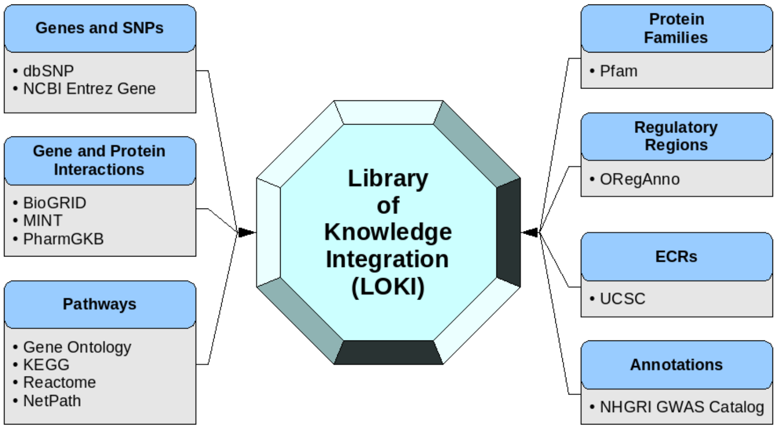

Knowledge Sources

Source	URL	Summary
**BioGRID**	http://thebiogrid.org	BioGRID is a repository with genetic and protein interaction data from model organisms and humans used by Biofilter for linking position and region data to interaction information.
**NCBI dbSNP**	http://www.ncbi.nlm.nih.gov /snp	A database of SNPs and multiple small-scale variations including insertions, deletions, microsatellites and non-polymorphic variants. This resource includes a complete list of known human SNPs and their base pair positions relative to the human reference genome. Biofilter uses the data of dbSNP in two ways: connecting SNP identifiers (RS numbers) of dbSNP to genomic positions and connecting retired identifiers to current identifiers.
**NCBI Gene**	http://www.ncbi.nlm.nih.gov /gene	Entrez is a search engine that allows users to search many discrete health sciences databases at the NCBI. The database provides an extensive list of known human genes, their beginning and ending base pair positions, and many alternate names and cross-referenced database identifiers. This data is used to connect gene symbols to their genomic regions, and to connect equivalent gene symbols and identifiers to each other.
**Gene Ontology**	http://www.geneontology.org	The Gene Ontology database defines terms representing gene product properties, such as cellular components, molecular function, and biological processes, within a hierarchical tree of ontology groups and related proteins.
**NHGRI GWAS Catalog**	http://www.genome.gov /gwastudies	The NHGRI GWAS Catalog provides associations between SNPs and various phenotypes which were discovered via genome-wide association studies (GWAS).
**MINT**	http://mint.bio.uniroma2.it /mint/Welcome.do	The Molecular Interaction database contains experimentally verified protein-protein interactions from the scientific literature, which are used in Biofilter for linking position and region data to interacting protein pairs.
**NetPath**	http://www.netpath.org	The NetPath database consists of curated human signaling pathways which are used by Biofilter.
**OregAnno**	http://www.oreganno.org /oregano	The Open REGulatory ANNOtation database is used by Biofilter for curation information about known regulatory elements from the scientific literature.
**Pfam**	http://pfam.sanger.ac.uk	The Pfam database is a large collection of protein families. The annotation of data respective to proteins within Biofilter is based on the information from Pfam.
**PharmGKB**	http://www.pharmgkb.org	Biofilture currently uses this database for pathway based data, future releases of Biofilter will also include gene-drug associations and pharmacological association study results.
**Reactome**	http://www.reactome.org /ReactomeGWT/entrypoint.html	Biofilter uses the information contained in Reactome to establish pathway and network relationships between genes.
**UCSC genome browser**	http://genome.ucsc.edu	This source provides access to a growing database of genomic sequence and annotations for a wide variety of organisms, currently we use the UCSC for location information for evolutionary conserved regions (ECRs) for Biofilter and to acess OregAnno's regulatory region data.

Data Types

Biofilter can work with and understand the relationships between six basic types of data:

SNP

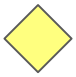

Specified by an RS number, i.e. "rs1234".
Used to refer to a known and documented SNP whose position can be retrieved from the knowledge database.

Position

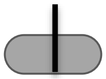

Specified by a chromosome and basepair location, i.e. "chr1:234".
Used to refer to any single genomic location, such as a single nucleotide polymorphism (SNP), single nucleotide variation (SNV), rare variant, or any other position of interest.

Region

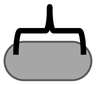

Specified by a chromosome and basepair range, i.e. "chr1:234-567".
Used to refer to any genomic region, such as a copy number variation (CNV), insertion/deletion (indel), gene coding region, evolutionarily conserved region (ECR), functional region, regulatory region, or any other region of interest.

Gene

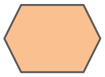

Specified by a name or other identifier, i.e. "A1BG" or "ENSG00000121410".
Used to refer to a known and documented gene, whose genomic region and associations with any pathways, interactions or other groups can be retrieved from the knowledge database.

Group

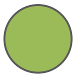

Specified by a name or other identifier, i.e. "lipid metabolic process" or "GO:0006629".
Used to refer to a known and documented pathway, ontological group, protein interaction, protein family, or any other grouping of genes, proteins or genomic regions that was provided by one of the external data sources.

Source

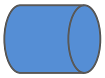

Specified by name, i.e. "GO".
Used to refer to a specific external data source.

Some of these data types are closely related, but behave in slightly different ways. For example a SNP and a position may be interchangeable in most cases, but not always: some RS numbers have no known genomic position while some have more than one, and any given genomic position could be associated with more than one RS number, or none at all. Similarly, some genes have no confirmed genomic region while some have several, and a given region might overlap or contain one gene, or many, or none.

The order in which these types have been listed is also significant: it is the sequence in which data can be cross-referenced within Biofilter. For example, a SNP (or RS number) and a gene have no direct relationship, but a SNP may have a known genomic position (or several), and that position may lie within a known region which is associated with a particular gene. To complete the chain, a gene may be associated with one or more groups of various types (interactions, pathways, etc.), and each of those groups was provided from a particular external data source.

Analysis Modes

Biofilter has three primary analysis modes which each make use of the available biological knowledge in slightly different ways.

Filtering

The most straightforward of Biofilter's primary functions is, as the name implies, filtering. Given any combination of input data, Biofilter can cross-reference the input data using the relationships stored in the knowledge database to generate a filtered dataset of any supported type (or types).

For example, a user can provide a list of SNPs (such as those covered by a genotyping platform) and a list of genes (such as those thought to be related to a particular phenotype) and request a filtered set of SNPs. Biofilter will use LOKI's knowledge of SNP positions and gene regions to filter the provided SNP list, removing all those that are not located within any of the provided genes.

The output data type does not necessarily have to be the same data type(s) provided as input. For example, a user can provide a list of SNPs and a list of groups and request the set of genes that match both lists. In this case, there is no input set of genes to use as a starting point so Biofilter will check all known genes found in the knowledge database. The result is a list of only the genes which include at least one of the specified SNPs, and are a part of at least one of the specified groups.

Finally, filtering is not limited to a single data type: Biofilter can also identify all of the unique combinations of data types which jointly meet the provided criteria. For example, given a list of SNPs and genes, Biofilter can produce a filtered set of SNP-gene pairs. The result is every combination of SNP and gene from the two lists where the SNP is within the gene.

Annotation

Biofilter can also annotate any of the supported data types with respect to any of the others. Like filtering, the annotations are based on the relationships stored in the knowledge database; unlike filtering, any data which cannot be annotated as requested (such as a SNP which is not located within any gene) will still be included in the output, with the annotation columns of the output simply left blank. Put another way, the difference between filtering and annotation is that filtering does not allow any blanks.

For example, a list of SNPs can be annotated with positions to generate a new list of all the same SNPs, but with extra columns containing the chromosome and genomic position for each SNP (if any). Any SNP with multiple known positions will be repeated, and any SNP with no known position will have blanks in the added columns.

Similarly, those same SNPs can be annotated with gene information; the result is similar, except that the added column contains the name of the gene containing the SNP's position. In this case a blank value can mean two things: either the SNP does not fall within any known gene region, or the SNP has no known position with which to search for gene regions.

Annotations can also be generated for combinations of data types, or for data types which were not provided as input. In these cases the annotation will be for the output of a filtering analysis. For example, suppose the user provides a list of SNPs and a list of groups, and then requests an annotation of genes to regions. Since no genes were provided as input, Biofilter will first perform a filtering analysis to identify all genes which contain at least one of the provided SNPs, and are also part of at least one of the provided groups. This filtered set of genes will then appear in the first column of the annotation output, followed by each gene's genomic region (if any).

Modeling

The last of Biofilter's primary analysis modes is a little different from filtering and annotation. In addition to simply cross-referencing any given data with the other available prior knowledge, Biofilter can also search for repeated patterns within the prior knowledge which might indicate the potential for important interactions between SNPs or genes.

The key idea behind this analysis is that any pathway, ontological category, protein family, experimental interaction, or other grouping of genes or proteins implies a relationship between each of those genes or proteins. If the same two genes appear together in more than one grouping, they're likely to have an important biological relationship; if they appear in multiple groups from several independent sources, then they're even more likely to be biologically related in some way. Biofilter has access to thousands of such groupings and can analyze all of them to identify the pairs of genes or SNPs appearing together in the greatest number of groupings and the widest array of original data sources. These pairs can then be tested for significance within a research dataset, avoiding the prohibitive computational and multiple-testing burden of an exhaustive pairwise analysis.

Biofilter can take any combination of input data and use it to focus the search for likely pairwise interaction models. For example, a user can provide a list of SNPs and request gene-gene models; Biofilter will then only consider models in which both genes contain at least one of the specified SNPs.

The models suggested by Biofilter are also ranked in order of likelihood, using an "implication index." This score is simply a combination of two tallies: the number of original data sources which contained the pair, and the number of different groups among those sources. For example a score of "2-3" indicates that the model appears in three different groups, and those groups originated with two different sources.

Since the interaction models are based on genes appearing together in multiple groups, Biofilter performs all model-building analyses by first generating gene-gene models. These baseline models can then be converted into models of any type by expanding each side independently, just like in a filtering analysis. For example if the user requests SNP models, Biofilter will take each baseline gene-gene model, separately map the two genes to all applicable SNPs, and then return all possible pairings between those two sets of SNPs.

Primary and Alternate Input Datasets

So far, the descriptions and examples of Biofilter's various analysis modes have implied that all user input exists in a single dataset. However, Biofilter can support two independent sets of user input data. These two datasets are used for slightly different things depending on the context, and so whenever

input data is provided to Biofilter, the user must specify which dataset it should be added to. For this purpose there are corresponding primary and alternate input options for each type of data: “SNP” and “ALT_SNP”, “REGION” and “ALT_REGION”, and so on.

In a filtering analysis, only the primary input dataset is used; any alternate input data has no effect.

In an annotation analysis, the primary and alternate input datasets are used separately on the two sides of the annotation. For example, if a user annotates SNPs with genes then the primary input data is used to limit which SNPs are annotated at all, while the alternate input data is used to limit which genes can be considered for annotation. Put another way, this means that if a SNP cannot be linked with the primary input data then it will not appear at all in the annotation output (even with blank annotation columns); likewise if a gene cannot be linked with the alternate input data then it will not appear as an annotation for any SNP, even if its genomic region does contain the SNP’s position.

In a modeling analysis, the primary and alternate input datasets are used similarly to annotation, with one extra option. By default, both parts of a model must match the primary input data in order for that model to be generated. If there is any alternate input data, then one of the two parts of the model must also match the alternate input. For example, the user could provide SNP list A as primary input and SNP list B as alternate input, and then request SNP models. Biofilter would then only generate SNP-SNP models in which both SNPs appear in list A, and at least one of them also appears in list B. With the ALTERNATE_MODEL_FILTERING option, the effect of the primary input is relaxed a bit so that it only applies to one part of the model, while the alternate input applies to the other. In this case, Biofilter would generate SNP-SNP models where one SNP is in list A and the other SNP is in list B.

Identifiers

Any given gene or group might go by many different names in different contexts, and Biofilter/LOKI accommodate this. For example, a single gene (let’s say “alpha-1-B glycoprotein”) might have one ID number assigned by NCBI’s Entrez Gene database (“1”), a different identifier assigned by Ensembl (“ENSG00000121410”), another one from HGNC (“5”), plus any number of symbolic abbreviations (*A1BG, A1B, ABG, GAB, HYST2477*).

Just as a single gene can have more than one name, there are also names which are known to be associated with more than one gene; these names are considered ambiguous. For example, although *A1B* is an alias of the gene *A1BG*, it is also an alias of the gene *SNTB1* (syntrophin, beta 1). Therefore if *A1B* appears in an input gene list file, Biofilter will not inherently recognize which gene the user intended to include. Likewise if *A1B* were to appear within the bulk biological data downloaded for LOKI, then Biofilter might not recognize which gene is actually part of some pathway.

Rather than attempting to compromise on a “one size fits all” approach to this ambiguity, Biofilter and LOKI support multiple interpretations of any ambiguity that was encountered while compiling the knowledge database. Each of these interpretations comes with a slightly different trade-off between false-positives and false-negatives, and the interpretation most appropriate to the task can be selected by the user at run-time. This is covered in greater detail in a later section, but it is important to bear in mind that ambiguity will be a part of relating and cross-referencing data across multiple independent sources. Biofilter’s results can change depending on the users choice for handling ambiguity.

Installation & Setup

Prerequisites

The following prerequisites are required to compile the LOKI database and run Biofilter:

- Python, version 2.7 or later
- Python module "apsw" (Another Python SQLite Wrapper)
- SQLite, version 3.6 or later

Note that the dependency on SQLite may be satisfied via the "apsw" Python module, since it often comes with an embedded copy of the necessary SQLite functionality. However, if LD Spline will be used (see below) then the SQLite development files will also be required, and these are not packaged with "apsw". In either case, if in doubt, consult your system administrator.

Platforms

Biofilter was developed in Python, and should therefore run on Linux, Mac OS X or Windows.

Installing Biofilter

Biofilter can be downloaded from www.ritchielab.psu.edu. To install it onto your system, simply use Python to run the included "setup.py" script with the "install" option:

```
python setup.py install
```

This will place the Biofilter and LOKI files in your system's usual place for Python-based software, which is typically alongside Python itself. The installation can also be done in a different location by using the "--prefix" or "--exec-prefix" options. If you wish to use LD profiles, add the "--ldprofile" option in order to compile and install ldspline (see **Appendix 2**).

Compiling Prior Knowledge

The LOKI prior knowledge database must be generated before Biofilter can be used. This is done with the "loki-build.py" script which was installed along with Biofilter. There are several options for this utility which are detailed below, but to get started, you just need "--knowledge" and "--update":

```
loki-build.py --knowledge loki.db --update
```

This will download and process the bulk data files from all supported knowledge sources, storing the result in the file "loki.db" (which we recommend naming after the current date, such as "loki-20130718.db"). The update process may take 4 to 8 hours depending on the speed of your internet connection, processor and filesystem, and requires up to 30 GB of free disk space: 10-20 GB of temporary storage ("C:\TEMP" on Windows, "/tmp" on Linux, etc) plus another 5-10 GB for the final knowledge database file.

By default, the LOKI build script will delete all sources' bulk data downloads after they have been processed. If the knowledge database will be updated frequently, it is recommended to keep these bulk files available so that any unchanged files will not need to be downloaded again. This can be accomplished with the "--archive" option.

LOKI Build Script Options

--help
Displays the program usage and immediately exits.

--version
Displays the software versions and immediately exits. Note that LOKI is built upon SQLite, which will also report its own software versions.

--knowledge Argument: <file> Default: *none*
Specifies the prior knowledge database file to use.

--archive Argument: <file> Default: *none*
Shorthand for specifying the same file as both the "--from-archive" and "--to-archive".

--from-archive Argument: <file> Default: *none*
An archive of downloaded bulk data from a previous run of the LOKI build script. The bulk data files available for download from each source will be compared against those found in the archive, and only files which have changed will be downloaded. If not specified, the script will start from scratch and download everything.

--to-archive Argument: <file> Default: *none*
A file in which to archive the downloaded bulk data for a later run of the LOKI build script. If not specified, the script will reclaim disk space by deleting all original data after processing it.

--temp-directory Argument: <directory> Default: *platform-dependent*
The directory in which to unpack the "--from-archive" (if any) and then download new bulk data. If not specified, the system's default temporary directory is used.

--list-sources Arguments: [source] […] Default: *none*
List the specified source module loaders' software versions and any options they accept. If no sources are specified, all available modules are listed.

--cache-only Argument: *none*
Causes the build script to skip checking any knowledge sources for available bulk data downloads, allowing it to function without an internet connection. Instead, only the files already available in the provided "--from-archive" file will be processed. If any source loader module is unable to find an expected file (such as if no archive was provided), that source loader will fail and no data will be updated for that source.

--***update*** Arguments: [source] […] Default: *all*
Instructs the build script to process the bulk data from the specified sources and update their representation in the knowledge database. If no sources are specified, all supported sources will be updated.

--***update-except*** Arguments: [source] […] Default: *none*
Similar to "--update" but with the opposite meaning for the specified sources: all supported sources will be updated **except** for the ones specified. If no sources are specified, none are excluded, and all supported sources are updated.

--***option*** Arguments: <source> <options> Default: *none*
Passes additional options to the specified source loader module. The options string must be of the form "option1=value,option2=value" for any number of options and values. Supported options and values for each source can be shown with "--list-sources".

--***finalize*** Argument: *none*
Causes the build script to discard all intermediate data and optimize the knowledge database (after performing an "--update", if any). This reduces the knowledge database file size and greatly improves its performance, however it will no longer be possible to update the file with any new source data.

--***verbose*** Argument: *none*
Prints additional informational messages to the screen.

--***test-data*** Argument: *none*
Switches the build script into test mode, in which it uses an alternate set of source loader modules. These sources do not contain actual biological knowledge; instead, they specify a minimal simulated set of knowledge which can be easily visualized and used to test and understand the functionality of LOKI and Biofilter. Knowledge database files created in test mode cannot be updated in the standard mode, and vice versa. Refer to the **Example Knowledge** section for more information.

Updating & Archiving Prior Knowledge

It is important to note that the various data sources integrated into LOKI can publish updated data at any time, according to their own schedules. This new data will not be available to Biofilter until the LOKI prior knowledge database is updated or regenerated. We recommend that users become familiar with how often the data sources are updated and plan to update LOKI accordingly, preferably at least once every few months.

If a given set of analyses need to be repeatable or verifiable, such as those published in a manuscript, we recommend storing an archived version of the LOKI knowledge database from the time of the analyses. These archived versions of the database can then be used to repeat or augment an analysis based on exactly the same prior knowledge, regardless of any updates that may have occurred in various data sources afterwards. For this purpose it may be useful to include the date in the filename of each newly compiled version of LOKI in order to carefully distinguish between older versions.

LD Profiles

Biofilter and LOKI allow for gene regions to be adjusted by the linkage disequilibrium (LD) patterns in a given population. When comparing a known gene region to any other region or position (such as CNVs or SNPs), areas in high LD with a gene can be considered part of the gene, even if the region lies outside of the gene's canonical boundaries.

LD profiles can be generated using LD Spline, a separate software tool bundled with Biofilter. For more information about LD Spline, please visit the *www.ritchielab.psu.edu* website; for details on generating and using LD profiles, see **Appendix 2**.

Using Biofilter

Biofilter can be run from a command-line terminal by executing “biofilter.py” (or “python biofilter.py”) and specifying the desired inputs, outputs and other optional settings. All options can either be provided directly on the command line (such as “biofilter.py --option-name”) or placed in one or more configuration files whose filenames are then provided on the command line (such as “biofilter.py analysis.config”). The former approach may be more convenient for setting up the necessary options to achieve the desired analysis, but the latter approach is recommended for any final runs, since the configuration file then serves as a record of exactly what was done. Any number of configuration files may be used, with options from later files overriding those from earlier files. Options on the command line override those from any configuration file.

The available options are the same no matter where they appear, but are formatted differently. Options on the command line are lower-case, start with two dashes and may contain single dashes to separate words (such as “--snp-file”), while in a configuration file the same option would be in upper-case, contain no dashes and instead use underscores to separate words (i.e. “SNP_FILE”). Many command line options also have alternative shorthand versions of one or a few letters, such as “-s” for “--snp-file” and “--aag” for “--allow-ambiguous-genes”.

All options are listed here in both their command line and configuration file forms. If an option allows or requires any further arguments, they are also noted along with their default values, if any. Arguments which are required are enclosed in <angle brackets>, while arguments which are optional are enclosed in [square brackets].

Many options have only two possible settings and therefore accept a single argument which can either be “yes” or “no” (or “on” or “off”, or “1” or “0”). Specifying these options with no argument is always interpreted as a “yes”, such that for example “VERBOSE yes” and “VERBOSE” have the same meaning. However, omitting such options entirely may default to either “yes” or “no” depending on the option.

Configuration Options

--help / HELP
Displays the program usage and immediately exits.

--version / VERSION
Displays the software versions and immediately exits. Note that Biofilter is built upon LOKI and SQLite, each of which will also report their own software versions.

--report-configuration / REPORT_CONFIGURATION
Argument: [yes/no] Default: no
Generates a Biofilter configuration file which specifies the current effective value of all program options, including any default options which were not overridden. This file can then be passed back in to Biofilter again in order to repeat exactly the same analysis.

--report-replication-fingerprint / REPORT_REPLICATION_FINGERPRINT

Argument: [yes/no] Default: no

When used along with REPORT_CONFIGURATION, this adds additional validation options to the resulting configuration file. These extra options specify all relevant software versions as well as a fingerprint of the data contained in the knowledge database file. When re-running a configuration file with these extra replication options, Biofilter will use them to ensure that neither Biofilter itself nor the LOKI knowledge database file have been updated since the original analysis; this in turn ensures that the re-run analysis will produce the same (or compatible) results as the original.

Prior Knowledge Options

--knowledge / KNOWLEDGE

Argument: <file> Default: *none*

Specifies the LOKI prior knowledge database file to use. If a relative path is provided it will be tried first from the current working directory, and then from the location of the Biofilter executable itself.

--report-genome-build / REPORT_GENOME_BUILD

Argument: [yes/no] Default: no

Displays the build version(s) of the human reference genome which was used as the basis for all genomic positions in the prior knowledge database (such as for SNP positions and gene regions). Any position or region data provided as input must be converted to the same build version in order to match correctly with the prior knowledge.

--report-gene-name-stats / REPORT_GENE_NAME_STATS

Argument: [yes/no] Default: no

Generates a report of the gene identifier types available in the knowledge database.

--report-group-name-stats / REPORT_GROUP_NAME_STATS

Argument: [yes/no] Default: no

Generates a report of the group identifier types available in the knowledge database.

--allow-unvalidated-snp-positions / ALLOW_UNVALIDATED_SNP_POSITIONS

Argument: [yes/no] Default: yes

Allows Biofilter to make use of all SNP-position mappings available in the knowledge database, even ones which the original data source identified as un-validated. When disabled, only validated positions are considered.

--allow-ambiguous-knowledge / ALLOW_AMBIGUOUS_KNOWLEDGE

Argument: [yes/no] Default: no

Allows Biofilter to make use of all potential gene-group mappings in the knowledge database, even if the gene was referred to with an ambiguous identifier. This will likely include some false-positive associations, but the alternative is likely to miss some true associations.

--reduce-ambiguous-knowledge / REDUCE_AMBIGUOUS_KNOWLEDGE

Argument: [no/implication/quality/any] Default: no

Enables a heuristic algorithm to attempt to resolve ambiguous gene-group mappings in the knowledge database. Providing this option with no argument is the same as using ‘any’, which applies all heuristic algorithms at once.

--report-ld-profiles / REPORT_LD_PROFILES

Argument: [yes/no] Default: no

Generates a report of the LD profiles available in the knowledge database. See Appendix 1 for details on generating LD profiles using LD Spline.

--ld-profile / LD_PROFILE

Argument: [ldprofile] Default: *none*

Specifies an alternate set of gene region boundaries which were pre-calculated by LD Spline to account for a population-specific linkage disequilibrium profile. When omitted or supplied with no argument, the default profile (containing the original unmodified gene boundaries) is used.

--verify-biofilter-version / VERIFY_BIOFILTER_VERSION

Argument: <version> Default: *none*

Ensure that the current version of Biofilter is the same as the one specified. This option is added automatically to configuration files generated with REPORT_REPLICATION_FINGERPRINT.

--verify-loki-version / VERIFY_LOKI_VERSION

Argument: <version> Default: *none*

Ensure that the current version of LOKI is the same as the one specified. This option is added automatically to configuration files generated with REPORT_REPLICATION_FINGERPRINT.

--verify-source-loader / VERIFY_SOURCE_LOADER

Arguments: <source> <version> Default: *none*

Ensure that the knowledge database file was generated with the specified version of a source data loader module. Can be used multiple times to specify versions for different sources. This option is added automatically to configuration files generated with REPORT_REPLICATION_FINGERPRINT.

--verify-source-option / VERIFY_SOURCE_OPTION

Arguments: <source> <option> <value> Default: *none*

Ensure that the knowledge database file was generated with the specified option value supplied to a source data loader module. Can be used multiple times to specify different options, or options for different sources. This option is added automatically to configuration files generated with REPORT_REPLICATION_FINGERPRINT.

--verify-source-file / VERIFY_SOURCE_FILE

Arguments: <source> <file> <date> <size> <md5> Default: *none*

Ensure that the knowledge database file was generated with the specified source data file. Can be used multiple times to specify different files, or files for different sources. This option is added automatically to configuration files generated with REPORT_REPLICATION_FINGERPRINT.

Primary Input Data Options

--snp / SNP

Arguments: <snp> [snp] […] Default: *none*

Adds (or intersects) the specified set of SNPs to (or with) the primary input dataset. SNPs must be provided as integer RS numbers with an optional “rs” prefix.

--snp-file / SNP_FILE

Arguments: <file> [file] […] Default: *none*

Adds (or intersects) the set of SNPs read from the specified files to (or with) the primary input dataset. Files must contain a single column formatted as in the SNP option.

--position / POSITION

Arguments: <position> [position] […] Default: *none*

Adds (or intersects) the specified set of positions to (or with) the primary input dataset. Positions must be provided as 2 to 4 fields separated by colons: “chr:pos”, “chr:label:pos” or “chr:label:ignored:pos”. Chromosomes may have an optional “chr” prefix.

--position-file / POSITION_FILE

Arguments: <file> [file] […] Default: *none*

Adds (or intersects) the set of positions read from the specified files to (or with) the primary input dataset. Files must contain 2 to 4 columns formatted as in the POSITION option, but separated by tabs instead of colons.

--region / REGION

Arguments: <region> [region] […] Default: *none*

Adds (or intersects) the specified set of regions to (or with) the primary input dataset. Regions must be provided as 3 or 4 fields separated by colons: “chr:start:stop” or “chr:label:start:stop”. Chromosomes may have an optional “chr” prefix.

--region-file / REGION_FILE

Arguments: <file> [file] […] Default: *none*

Adds (or intersects) the set of regions read from the specified files to (or with) the primary input dataset. Files must contain 3 or 4 columns formatted as in the REGION option, but separated by tabs instead of colons.

--gene / GENE

Arguments: <gene> [gene] […] Default: *none*

Adds (or intersects) the specified set of genes to (or with) the primary input dataset. The specified genes will be interpreted according to the GENE_IDENTIFIER_TYPE option.

--gene-file / GENE_FILE

Arguments: <file> [file] […] Default: *none*

Adds (or intersects) the set of genes read from the specified files to (or with) the primary input dataset. Files must contain 1 or 2 columns separated by tabs. For 1-column files, genes are interpreted according to the GENE_IDENTIFIER_TYPE option. For 2-column files, the first column specifies the gene identifier type by which the second column will be interpreted.

--gene-identifier-type / GENE_IDENTIFIER_TYPE

Argument: [type] Default: -

Specifies the identifier type with which to interpret all input gene identifiers. If no type or an empty type is provided, all possible types are tried for each identifier. If the special type “-“ is provided (the default), identifiers are interpreted as primary gene labels.

--allow-ambiguous-genes / ALLOW_AMBIGUOUS_GENES

Argument: [yes/no] Default: no

When enabled, any input gene identifier which matches multiple genes will be interpreted as if all of those genes had been specified. When disabled (the default), ambiguous gene identifiers are ignored.

--gene-search / GENE_SEARCH

Argument: text Default: *none*

Adds (or intersects) the matching set of genes to (or with) the primary input dataset. Matching genes are identified by searching for the provided text in all labels, descriptions and identifiers associated with each known gene.

--group / GROUP

Arguments: <group> [group] […] Default: *none*

Adds (or intersects) the specified set of groups to (or with) the primary input dataset. The specified groups will be interpreted according to the GROUP_IDENTIFIER_TYPE option.

--group-file / GROUP_FILE

Arguments: <file> [file] […] Default: *none*

Adds (or intersects) the set of groups read from the specified files to (or with) the primary input dataset. Files must contain 1 or 2 columns separated by tabs. For 1-column files, genes are interpreted according to the GENE_IDENTIFIER_TYPE option. For 2-column files, the first column specifies the gene identifier type by which the second column will be interpreted.

--group-identifier-type / GROUP_IDENTIFIER_TYPE

Argument: [type] Default: -

Specifies the identifier type with which to interpret all input group identifiers. If no type or an empty type is provided, all possible types are tried for each identifier. If the special type "-" is provided (the default), identifiers are interpreted as primary group labels.

--allow-ambiguous-groups / ALLOW_AMBIGUOUS_GROUPS

Argument: [yes/no] Default: no

When enabled, any input group identifier which matches multiple groups will be interpreted as if all of those groups had been specified. When disabled (the default), ambiguous group identifiers are ignored.

--group-search / GROUP_SEARCH

Argument: text Default: *none*

Adds (or intersects) the matching set of groups to (or with) the primary input dataset. Matching groups are identified by searching for the provided text in all labels, descriptions and identifiers associated with each known group.

--source / SOURCE

Arguments: <source> [source] […] Default: *none*

Adds (or intersects) the specified set of sources to (or with) the primary input dataset.

--source-file / SOURCE_FILE

Arguments: <file> [file] […] Default: *none*

Adds (or intersects) the set of sources read from the specified files to (or with) the primary input dataset.

Alternate Input Data Options

--alt-snp / ALT_SNP

Arguments: <snp> [snp] […] Default: *none*

Adds (or intersects) the specified set of SNPs to (or with) the alternate input dataset. SNPs must be provided as integer RS numbers with an optional "rs" prefix.

--alt-snp-file / ALT_SNP_FILE

Arguments: <file> [file] […] Default: *none*

Adds (or intersects) the set of SNPs read from the specified files to (or with) the alternate input dataset. Files must contain a single column formatted as in the SNP option.

--alt-position / ALT_POSITION

Arguments: <position> [position] […] Default: *none*

Adds (or intersects) the specified set of positions to (or with) the alternate input dataset. Positions must be provided as 2 to 4 fields separated by colons: "chr:pos", "chr:label:pos" or "chr:label:ignored:pos". Chromosomes may have an optional "chr" prefix.

--alt-position-file / ALT_POSITION_FILE

Arguments: <file> [file] […] Default: *none*

Adds (or intersects) the set of positions read from the specified files to (or with) the alternate input dataset. Files must contain 2 to 4 columns formatted as in the POSITION option, but separated by tabs instead of colons.

--alt-region / ALT_REGION

Arguments: <region> [region] […] Default: *none*

Adds (or intersects) the specified set of regions to (or with) the alternate input dataset. Regions must be provided as 3 or 4 fields separated by colons: "chr:start:stop" or "chr:label:start:stop". Chromosomes may have an optional "chr" prefix.

--alt-region-file / ALT_REGION_FILE

Arguments: <file> [file] […] Default: *none*

Adds (or intersects) the set of regions read from the specified files to (or with) the alternate input dataset. Files must contain 3 or 4 columns formatted as in the REGION option, but separated by tabs instead of colons.

--alt-gene / ALT_GENE

Arguments: <gene> [gene] […] Default: *none*

Adds (or intersects) the specified set of genes to (or with) the alternate input dataset. The specified genes will be interpreted according to the GENE_IDENTIFIER_TYPE option.

--alt-gene-file / ALT_GENE_FILE

Arguments: <file> [file] […] Default: *none*

Adds (or intersects) the set of genes read from the specified files to (or with) the alternate input dataset. Files must contain 1 or 2 columns separated by tabs. For 1-column files, genes are interpreted according to the GENE_IDENTIFIER_TYPE option. For 2-column files, the first column specifies the gene identifier type by which the second column will be interpreted.

--alt-gene-search* / *ALT_GENE_SEARCH

Argument: text Default: *none*

Adds (or intersects) the matching set of genes to (or with) the alternate input dataset. Matching genes are identified by searching for the provided text in all labels, descriptions and identifiers associated with each known gene.

--alt-group* / *ALT_GROUP

Arguments: <group> [group] […] Default: *none*

Adds (or intersects) the specified set of groups to (or with) the alternate input dataset. The specified groups will be interpreted according to the GROUP_IDENTIFIER_TYPE option.

--alt-group-file* / *ALT_GROUP_FILE

Arguments: <file> [file] […] Default: *none*

Adds (or intersects) the set of groups read from the specified files to (or with) the alternate input dataset. Files must contain 1 or 2 columns separated by tabs. For 1-column files, genes are interpreted according to the GENE_IDENTIFIER_TYPE option. For 2-column files, the first column specifies the gene identifier type by which the second column will be interpreted.

--alt-group-search* / *ALT_GROUP_SEARCH

Argument: text Default: *none*

Adds (or intersects) the matching set of groups to (or with) the alternate input dataset. Matching groups are identified by searching for the provided text in all labels, descriptions and identifiers associated with each known group.

--alt-source* / *ALT_SOURCE

Arguments: <source> [source] […] Default: *none*

Adds (or intersects) the specified set of sources to (or with) the alternate input dataset.

--alt-source-file* / *ALT_SOURCE_FILE

Arguments: <file> [file] […] Default: *none*

Adds (or intersects) the set of sources read from the specified files to (or with) the alternate input dataset.

Positional Matching Options

--region-position-margin* / *REGION_POSITION_MARGIN

Argument: <bases> Default: 0

Defines an extra margin beyond the boundaries of all genomic regions within which a position will still be considered a match with the region. With no suffix or a "b" suffix the margin is interpreted as basepairs; with a "kb" or "mb" suffix it is measured in kilobases or megabases, respectively.

--region-match-percent* / *REGION_MATCH_PERCENT

Argument: <percentage> Default: 100

Defines the minimum proportion of overlap between two regions in order to consider them a match. The percentage is measured in terms of the shorter region, such that 100% overlap always implies one region equal to or completely contained within the other. When combined with REGION_MATCH_BASES, both requirements are enforced independently. For this reason, the default value for REGION_MATCH_PERCENT is ignored if REGION_MATCH_BASES is used alone.

--region-match-bases* / *REGION_MATCH_BASES

Argument: <bases> Default: 0

Defines the minimum number of basepairs of overlap between two regions in order to consider them a match. With no suffix or a "b" suffix the overlap is interpreted as basepairs; with a "kb" or "mb" suffix it is measured in kilobases or megabases, respectively. When combined with REGION_MATCH_PERCENT, both requirements are enforced independently.

Model Building Options

--maximum-model-count* / *MAXIMUM_MODEL_COUNT

Argument: <count> Default: 0

Limits the number of models that will be generated, in order to reduce processing time. A value of 0 (the default) means no limit.

--alternate-model-filtering* / *ALTERNATE_MODEL_FILTERING

Argument: [yes/no] Default: no

When enabled, the primary input dataset is only applied to one side of a generated model, while the alternate input dataset is applied to the other. When disabled (the default), the primary input dataset applies to both sides of each model.

--all-pairwise-models* / *ALL_PAIRWISE_MODELS

Argument: [yes/no] Default: no

When enabled, model generation results in all possible pairwise combinations of data which conform to the primary and alternate input datasets. Note that this means the models have no score or ranking, since the prior knowledge is not searched for patterns. When disabled (the default), models are only generated which are supported by one or more groupings within the prior knowledge database.

--maximum-model-group-size* / *MAXIMUM_MODEL_GROUP_SIZE

Argument: <size> Default: 30

Limits the size of a grouping in the prior knowledge which can be used as part of a model generation analysis; any group which contains more genes than this limit is ignored for purposes of model generation. A value of 0 means no limit.

--minimum-model-score* / *MINIMUM_MODEL_SCORE

Argument: <score> Default: 2

Sets the minimum source-tally score for generated model; a model must be supported by groups from at least this many sources in order to be returned.

--sort-models* / *SORT_MODELS

Argument: [yes/no] Default: yes

When enabled (the default), models are output in descending order by score. When combined with MAXIMUM_MODEL_COUNT, this guarantees that only the highest-scoring models are output. When disabled, models are output in an unpredictable order.

Output Options

--quiet / QUIET

Argument: [yes/no] Default: no

When enabled, no warnings or informational messages are printed to the screen. However, all information is still written to the log file, and certain unrecoverable errors are still printed to the screen.

--verbose / VERBOSE

Argument: [yes/no] Default: no

When enabled, informational messages are printed to the screen in addition to warnings and errors.

--prefix / PREFIX

Argument: <prefix> Default: "biofilter"

Sets the prefix for all output filenames, which is then combined with a unique suffix for each type of output. The prefix may contain an absolute or relative path in order to write output to a different directory.

--overwrite / OVERWRITE

Argument: [yes/no] Default: no

Allows Biofilter to erase and overwrite any output file which already exists. When disabled (the default), Biofilter exits with an error to prevent any existing files from being overwritten.

--stdout / STDOUT

Argument: [yes/no] Default: no

Causes all output data to be written directly to the screen rather than saved to a file. On most platforms this output can then be sent directly into another program.

--report-invalid-input / REPORT_INVALID_INPUT

Argument: [yes/no] Default: no

Causes any input data which was not understood by Biofilter to be copied into a separate output report file. This file also includes comments describing the error with each piece of data.

--filter / FILTER

Argument: <type> [type] […] Default: *none*

Perform a filtering analysis which outputs the specified type(s). If a single type is requested, the output will be in exactly the same format that Biofilter requires as input for that data type; additional types are simply appended left-to-right in the order requested.

--annotate / ANNOTATE

Argument: <type> [type] […] [:] <type> [type] […] Default: *none*

Perform an annotation analysis which outputs the specified type(s). The starting point for the annotation is the first specified type (or, if a colon is used, the combination of types before the colon); all additional types are optional and will be left blank if no suitable match can be found.

--model / MODEL

Argument: <type> [type] […] [:] [type] […] Default: *none*

Perform a modeling analysis which generates models of the specified type(s). If a colon is used, the types before and after the colon will appear on the left and right sides of the generated models, respectively; with no colon, both sides of the models will have the same type(s).

Input File Formats

For all input files in Biofilter, lines beginning with the symbol "#" will be ignored. This is useful for placing comments within input files that will not be a part of the analysis.

Configuration Files

Any option which can be used on the command line can also be used in a configuration file. Each option must appear as the first item on a line, and any arguments to that option must be separated by whitespace (any number of tabs or spaces).

If an argument to an option must itself contain spaces (for example a multi-word gene or group identifier), the argument may be enclosed with "double quotes" to prevent the additional words in the argument from being interpreted as a separate arguments. If an argument must itself contain double quotes, they must be escaped with a backslash, \"like so\".

There is also one extra option which may only be used in a configuration file: INCLUDE. This option requires one or more filename arguments and causes Biofilter to read each specified file as an additional configuration file. Included files are processed in full before any other options in the original configuration file. For example, if file A includes file B and both files specify the same option, then the option's setting or value from file A will always override the one from file B, even if it appears before the INCLUDE instruction. Included configuration files may also include further files; there is no limit to this recursion, except that any loops (i.e. A includes B which includes A) will raise an error.

This example configuration file was generated by the REPORT_CONFIGURATION option, with everything else left at default values:

```
# Biofilter configuration file
#   generated Thu, 18 Jul 2013 12:00:00
#   Biofilter version 2.1.0 (2013-07-18)
#   LOKI version 2.1.0 (2013-07-18)

REPORT_CONFIGURATION                yes
REPORT_REPLICATION_FINGERPRINT      no
REPORT_GENOME_BUILD                 no
REPORT_GENE_NAME_STATS              no
REPORT_GROUP_NAME_STATS             no
ALLOW_UNVALIDATED_SNP_POSITIONS     yes
ALLOW_AMBIGUOUS_KNOWLEDGE           no
REDUCE_AMBIGUOUS_KNOWLEDGE          no
GENE_IDENTIFIER_TYPE                -
ALLOW_AMBIGUOUS_GENES               no
GROUP_IDENTIFIER_TYPE               -
ALLOW_AMBIGUOUS_GROUPS              no
REGION_POSITION_MARGIN              0
REGION_MATCH_PERCENT                100.0
REGION_MATCH_BASES                  0
```

```
MAXIMUM_MODEL_COUNT                    0
ALTERNATE_MODEL_FILTERING              no
ALL_PAIRWISE_MODELS                    no
MAXIMUM_MODEL_GROUP_SIZE               30
MINIMUM_MODEL_SCORE                    2
SORT_MODELS                            yes
QUIET                                  no
VERBOSE                                no
PREFIX                                 biofilter
OVERWRITE                              no
STDOUT                                 no
REPORT_INVALID_INPUT                   no
```

SNP List Input Files

SNP input files only require one column listing the RS number of each SNP, which may optionally begin with the "rs" prefix. If all inputs and outputs only deal with SNPs, then these RS numbers will all be used as-is.

If any part of the analysis involves any other data types, however, then the provided RS numbers will have to be mapped to positions using the prior knowledge database. In this case a single RS number may correspond to multiple genomic positions, or it may have no known position (at least on the current genomic reference build). For these reasons it may be preferable to provide positions directly, if available, rather than relying on SNP identifiers.

Example:

```
#snp
rs123
456
rs789
```

Position Data Input Files

The input file format for position data is similar to the MAP file format used in PLINK (pngu.mgh.harvard.edu/~purcell/plink/data.shtml#map). Up to four columns are allowed, separated by tab characters:

- Chromosome (1-22, X, Y, MT)
- RS number or other label
- Genetic distance (ignored by Biofilter)
- Base pair position

Since the genetic distance column is not used by Biofilter, it may be omitted entirely for a three-column format (equivalent to PLINK's --map3 option). The label column may also be omitted for a two-column format including only the chromosome and position; in this case a label of the form

“chr1:2345” will be automatically generated. Note that if the label column is used, it does not necessarily have to be a known SNP’s RS number; whatever arbitrary label is provided will be used by Biofilter to refer to the position whenever it appears in any output file.

Example:

```
#chr   label   pos
7      rs123   24966446
7      rs456   24962419
3      rs789   29397015
```

Region Data Input Files

The file format for region input data is similar to that of positional data. Up to four columns are allowed, separated by tab characters:

- Chromosome (1-22, X, Y, MT)
- Gene symbol or other label
- Base pair start position
- Base pair stop position

As with positional data, the label column does not necessarily have to be a known gene symbol, and can be omitted entirely. If the column is omitted then a label of the form “chr1:2345-6789” will be generated automatically; if labels are provided, then Biofilter will use them to refer to the regions whenever they appear in any output file.

Example:

```
#chr   label    start      stop
7      THSD7A   11410061   11871823
7      OSBPL3   24836158   25019759
3      RBMS3    29322802   30051885
```

Gene and Group List Input Files

Like the SNP input file format, a gene or group input file may simply be a single column of identifiers. Unlike the SNP file format, gene or group input files may alternatively include two columns separated by a tab character; in this case, the first column lists the type of the identifier which is in the second column on the same line.

The GENE_IDENTIFIER_TYPE and GROUP_IDENTIFIER_TYPE options specify the default type for any user-provided gene or group identifiers, respectively. This applies to any identifiers given directly via the GENE or GROUP options, and any identifiers listed in single-column gene or group list input files. These options do not apply to two-column gene or group input files, since those files specify their own identifier types in the first column.

An empty identifier type (a blank in the first column of a two-column gene input file, or a GENE/GROUP_IDENTIFIER_TYPE option with no argument) causes Biofilter to attempt to interpret the identifier using any known type. The special identifier type "-" instead causes Biofilter to interpret identifiers as primary labels of genes or groups, and the special type "=" accepts the gene_id or group_id output values from a previous Biofilter run.

It is important to recall that gene and group identifiers can vary in their degree of uniqueness. For analyses that depend on a gene's genomic region (such as comparisons with SNPs or other positions) it may be preferable to provide the regions directly rather than relying on gene identifiers. If a single identifier matches more than one gene or group, Biofilter will ignore it unless the appropriate ALLOW_AMBIGUOUS_GENES or ALLOW_AMBIGUOUS_GROUPS option is used.

Examples:

```
#gene
THSD7A
OSBPL3
RBMS3
```

```
#namespace      name
symbol          THSD7A
entrez_gid      26031
ensemble_gid    ENSG00000144642
```

Source List Input Files

Since the knowledge sources in LOKI all have single, unique names, there are no identifier types to consider. Source input files simply contain a single column with the name of a source on each line.

Note that sources play a slightly different role in Biofilter than in LOKI. When building the prior knowledge database, every source is relevant because they all contribute a different set of knowledge to the final product: many sources provide groupings of genes or proteins (pathways, interactions, etc), while others provide information about genes or SNPs themselves (such as their regions or boundaries, alternate names, etc). In Biofilter, however, sources are only considered in connection with groups; providing a source list to focus a Biofilter analysis is therefore exactly the same as providing a group list which includes every group from the source(s) in the source list. In particular, the sources which LOKI used to define basic SNP and gene information (such as "dbsnp" or "entrez") are not relevant to Biofilter since those sources generally do not define any groupings of genes; consequently, using any of those sources as inputs to Biofilter will generally result in no output.

Example:

```
#source
netpath
```

Output File Formats

Configuration Report

The format of a configuration output file is, by design, identical to a configuration input file. The details of that format can be found in the corresponding section of the previous chapter.

Note however that the INCLUDE instruction is not relevant for configuration output files because the structure of inclusions is not preserved internally. This means that even if the configuration file(s) provided to Biofilter include other configuration files, the report generated by the REPORT_CONFIGURATION option will not contain any INCLUDE instructions. Instead, all options from all included files will be merged into a single reported configuration.

Gene and Group Name Statistics Reports

These reports list all of the types of identifiers available for genes or groups, respectively, along with some statistics about their overall uniqueness. For example, this is the gene name statistics report at the time of writing:

```
#type           names     unique    ambiguous
symbol          91687     89417     2270
entrez_gid      62977     62977     0
refseq_gid      48880     48880     0
refseq_pid      35719     35719     0
ensembl_gid     50159     50098     61
ensembl_pid     31225     31225     0
hgnc_id         33036     33036     0
mim_id          15446     15446     0
hprd_id         18065     18065     0
vega_id         17572     17545     27
rgd_id          268       268       0
mirbase_id      1523      1523      0
unigene_gid     25016     24131     885
uniprot_gid     101047    101047    0
uniprot_pid     105084    98839     6245
pharmgkb_gid    27062     27062     0
```

The labels in the first column are the identifier types themselves; these are the values which can be used with the GENE_IDENTIFIER_TYPE option or in the first column of a two-column gene list input file. The second column shows the total number of distinct identifiers of that type which are found in the prior knowledge database file; for example, there are 91,687 different “symbol” identifiers, which are symbolic abbreviations of genes (i.e. “A1BG”). The second and third columns break that total down into the number which are associated with only one gene (unique identifiers) and the number which are associated with multiple genes (ambiguous identifiers).

The names of the identifier types are defined by LOKI, and generally correspond to the organization or project which assigns that type of name, followed by the particular kind of thing being named. For

example “entrez_gid” refers to the numeric gene numbers assigned by NCBI’s Entrez Gene database, while “ensembl_pid” refers to protein identifiers assigned by Ensembl.

LD Profiles Report

This report lists the LD profiles available in the knowledge database. If LD Spline has not been used to calculate LD-adjusted gene boundaries, then only the default profile with canonical gene boundaries will be shown.

Invalid Input Reports

If the REPORT_INVALID_INPUT option has been enabled, then any user input data which cannot be parsed or understood by Biofilter will appear in one of these report files. A separate file is generated for each type of input (SNP, position, region, etc.), and for each invalid input line, that entire line will be copied to the corresponding report file preceded by a comment line describing the error. For example, the SNP input file on the left will yield the invalid SNP report file on the right:

#snp<br>rs12<br>rs34<br>chr5:678<br>rs90	# invalid literal for long() with base 10: 'chr5:678'<br>chr5:678

One of the inputs was not understood as a valid RS number, but the other three were parsed successfully and added to the input dataset.

Analysis Outputs

Filtering, annotation and modeling analyses always return one or more tab-separated columns, but the number and contents of those columns can vary. Each analysis mode allows the user to exactly specify the desired output columns.

In the simplest case, the user can request one of the six data types which Biofilter also takes as input: SNP, position, region, gene, group or source. The output will then contain one or more columns describing the specified data type, in exactly the same format as Biofilter requires for input of the same type. For example, SNP output produces a single column of RS numbers, position output produces three columns (chromosome, label, position), and so on. More than one basic type can also be output together (and is required for annotation and modeling analyses), in which case the columns corresponding to any additional types are simply appended in order to the final output. For example, the analysis options on the left will produce the output columns on the right:

FILTER position	chr position pos
FILTER gene snp	gene snp
ANNOTATE gene region	gene chr region start stop
MODEL gene	gene1 gene2 score

Note that there are two different places Biofilter could draw from when outputting any given type of

data: one of the user input datasets, or the prior knowledge database. If an output type is requested which was not provided as input then the choice is clear, and Biofilter will produce the requested output based on the data contained in the knowledge database. For any data type which was provided as input, however, Biofilter will pull any corresponding output columns from the input data rather than the knowledge database.

This means, for example, that if regions are supplied as input and both “gene” and “region” are requested as output, then the result may not be as expected. The output will list both genes and regions, and the genes in the first column will indeed be the ones whose genomic region matched one of the provided input regions. However, the region shown next to each gene will *not* be that gene’s region, as one might hope; it will instead be the user-provided input region which matched the gene’s region.

Biofilter provides additional output options to deal with situations such as these. The six basic types will suffice for most use cases, but they are actually only shorthand for their respective sets of individual output columns. For more particular use cases there are a few additional shorthand types (such as “generegion”), and any single output column may also be requested individually. This includes each separate column from any of the six data type outputs (such as “region_chr” which is the first of four columns included in the “region” output type), as well as some columns which are not included in any of the shorthand sets.

Biofilter currently supports the following outputs:

`snp`	Shorthand for: snp_label
`snp_id`	The SNP’s RS number, with no prefix; if an input SNP was merged, the current (new) RS number is shown
`snp_label`	The SNP’s RS number, with “rs” prefix; if an input SNP was merged, the user-provided (old) RS number is shown
`position`	Shorthand for: position_chr , position_label , position_pos
`position_id`	An arbitrary unique ID number for the position; can be used to distinguish unlabeled positions with identical genomic locations
`position_label`	The provided (or generated) label for an input position, or the RS number (with “rs” prefix) for a SNP position from the knowledge database
`position_chr`	The position’s chromosome number or name
`position_pos`	The position’s basepair location
`region`	Shorthand for: region_chr , region_label , region_start , region_stop
`region_id`	An arbitrary unique ID number for the region; can be used to distinguish unlabeled regions with identical genomic start and stop locations
`region_label`	The provided (or generated) label for an input region, or the primary label for a region from the knowledge database
`region_chr`	The region’s chromosome number or name
`region_start`	The region’s basepair start location
`region_stop`	The region’s basepair stop location

generegion	Shorthand for: region_chr , gene_label , region_start , region_stop Similar to "region" except that only gene regions from the knowledge database are returned, even if the user also provided input regions
gene	Shorthand for: gene_label
gene_id	An arbitrary unique ID number for the gene; can be used to distinguish genes with identical labels
gene_label	The provided identifier for an input gene, or the primary label for a gene from the knowledge database
gene_description	The gene's descriptive text from the knowledge database, if any
gene_identifiers	All known identifiers for the gene, of any type; formatted as "type:name|type:name|…"
gene_symbols	All known "symbol"-type identifiers (symbolic aliases) for the gene, formatted as "symbol|symbol|…"
upstream	Shorthand for: upstream_label , upstream_distance
upstream_id	An arbitrary unique ID number for the closest upstream gene
upstream_label	The primary label for the closest upstream gene
upsteam_distance	The distance to the closest upstream gene
upsteam_start	The closest upstream gene's basepair start location
upsteam_stop	The closest upstream gene's basepair stop location
downstream	Shorthand for: downstream_label , downstream_distance
downstream_id	An arbitrary unique ID number for the closest downstream gene
downstream_label	The primary label for the closest downstream gene
downstream_distance	The distance to the closest downstream gene
downstream_start	The closest downstream gene's basepair start location
downstream_stop	The closest downstream gene's basepair stop location
group	Shorthand for: group_label
group_id	An arbitrary unique ID number for the group; can be used to distinguish groups with identical labels
group_label	The provided identifier for an input group, or the primary label for a group from the knowledge database
group_description	The group's descriptive text from the knowledge database, if any
group_identifiers	All known identifiers for the group, of any type; formatted as "type:name|type:name|…"
source	Shorthand for: source_label
source_id	An arbitrary unique ID number for the source; included for completeness
source_label	The source's name
gwas	Shorthand for: gwas_trait , gwas_snps , gwas_orbeta , gwas_allele95ci , gwas_riskAfreq , gwas_pubmed
gwas_rs	The RS# which led to the GWAS annotation match
gwas_chr	The chromosome on which the GWAS match was found
gwas_pos	The basepair location at which the GWAS match was found
gwas_trait	The GWAS annotation's associated trait or phenotype
gwas_snps	The full list of SNPs in the GWAS association
gwas_orbeta	The odds ratio or beta of the GWAS association
gwas_allele95ci	The allele 95% confidence interval of the GWAS association
gwas_riskAfreq	The risk allele frequency of the GWAS association
gwas_pubmed	The PubMedID of the GWAS association

Inspection of Biofilter's source code may reveal additional supported columns. They are not documented here because they are only used for internal or debugging purposes and may change or disappear in a future release; use them at your own risk.

Example Knowledge

In order to provide examples of filtering, annotation, and model building commands for Biofilter 2.0, we have provided a simulated LOKI database. This simulated database contains three fictitious sources (named "light", "paint" and "spectrum") which define eleven pathways (named "red", "green", "blue", "gray", "cyan", "magenta", "yellow", "gray", "orange", "indigo", "violet"), linked to 13 genes and 21 SNPs.

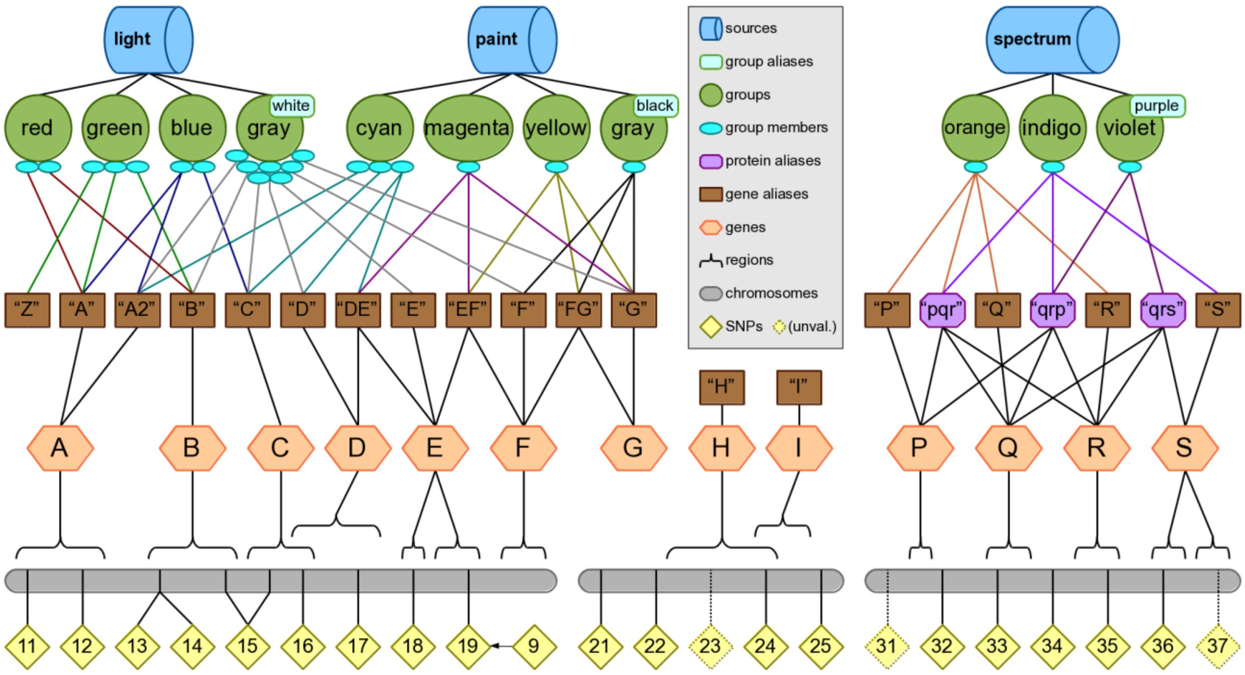

This simulated knowledge is intended to provide easily-understood examples of Biofilter's functionality without relying on real-world cases which might become outdated. Many important concepts and edge cases are represented here, such as two groups with the same primary label ("gray") which can only be differentiated by their aliases ("white" and "black"), some genes with multiple aliases (i.e. "A" and "A2"), and some aliases referring to multiple genes (i.e. "DE" could be gene D or gene E).

The groups from the "paint" and "spectrum" sources demonstrate many varieties of ambiguity. These are discussed in depth in **Appendix 1**, but for the examples in this chapter we will assume strict ambiguity options. We can then simplify the diagram of the knowledge by showing associations between groups and genes without the messy intermediate layer of aliases; in the resulting diagram below, the dotted lines indicate associations which will be ignored by default, but may appear if the ambiguity settings are changed.

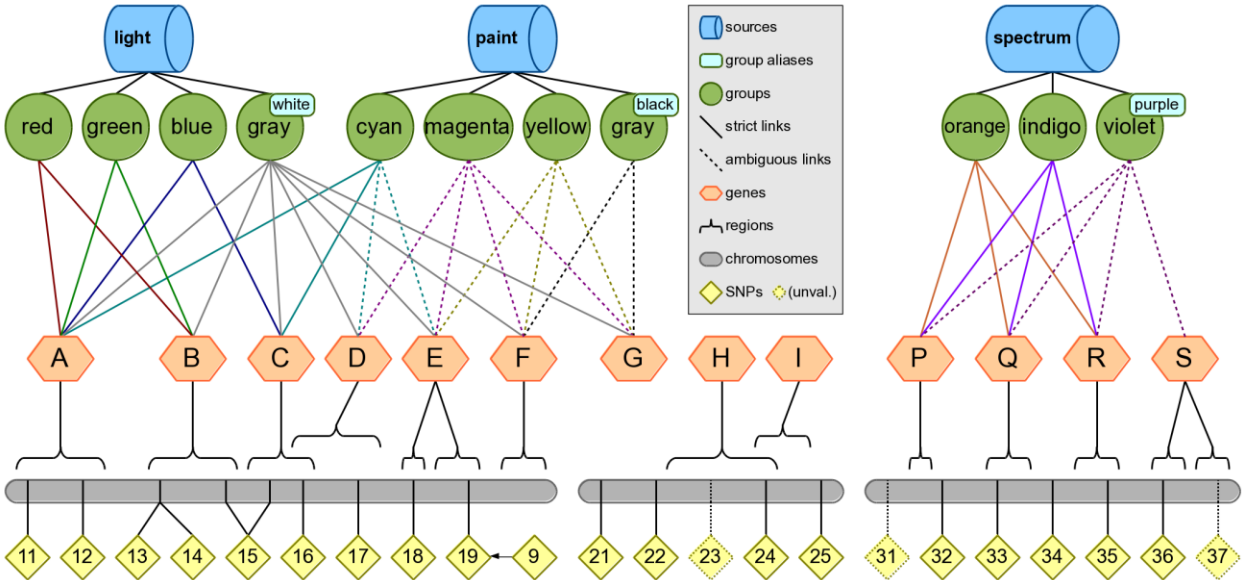

In order to reproduce the following examples using your own copy of Biofilter, you must run the "loki-build.py" script using the "--test-data" option; refer to the **Installation & Setup** section for details.

Example Commands

Filtering Examples

Example 1: Filtering a list of SNPs by a genotyping platform, where input1 is the first list of SNPs and input2 is the list of SNPs on the genotyping platform.

Input files:

**input1**	**input2**
#snp<br>rs9<br>rs11<br>rs12<br>rs13<br>rs14<br>rs15<br>rs16	#snp<br>rs14<br>rs15<br>rs16<br>rs17<br>rs18<br>rs19

Configuration:

```
KNOWLEDGE test.db
SNP_FILE input1
SNP_FILE input2
FILTER snp
```

Output:

```
#snp
rs9
rs14
rs15
rs16
```

Note: The lists of input SNPs are checked against a dbSNP list of SNP ID's that have been merged, and any outdated RSIDs are updated with the new RSID. In the example knowledge, rs9 has been merged into rs19; this is why rs9 appears in the output.

Example 2: Output a list of SNPs from a genotyping platform that correspond to a list of genes.

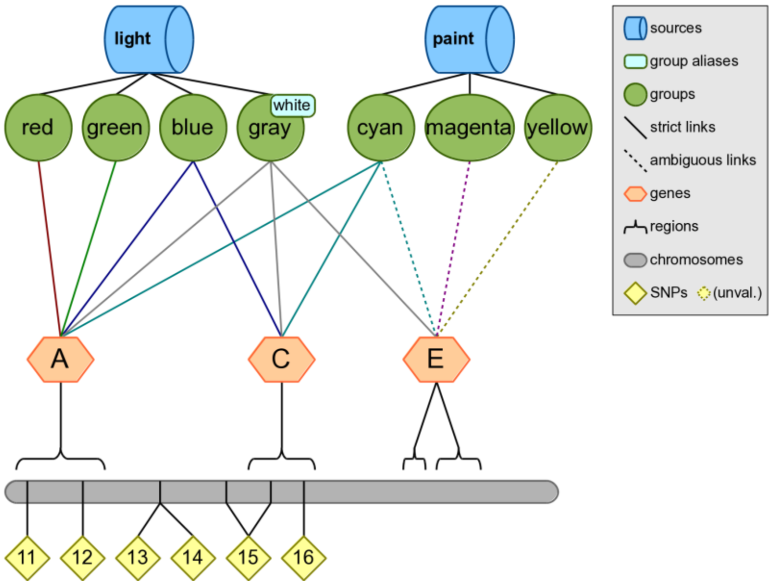

Input files:

**input1**	**input2**
#snp<br>rs11<br>rs12<br>rs13<br>rs14<br>rs15<br>rs16	#gene<br>A<br>C<br>E

Configuration:

```
KNOWLEDGE test.db
SNP_FILE input1
GENE_FILE input2
FILTER snp
```

Output:

```
#snp
rs11
rs12
rs15
rs16
```

Example 3: Input a list of groups, output regions within those groups.

Configuration:

```
KNOWLEDGE test.db
GROUP red green cyan magenta orange indigo
FILTER region
```

Output:

```
#chr  region  start  stop
1     A       8      22
1     B       28     52
1     C       54     62
3     P       14     18
3     Q       28     36
3     R       44     52
```

Example 4: Output a list of all genes within a data source.

Configuration:

```
KNOWLEDGE test.db
SOURCE light
FILTER gene
```

Output:

```
#gene
A
B
C
D
E
F
G
```

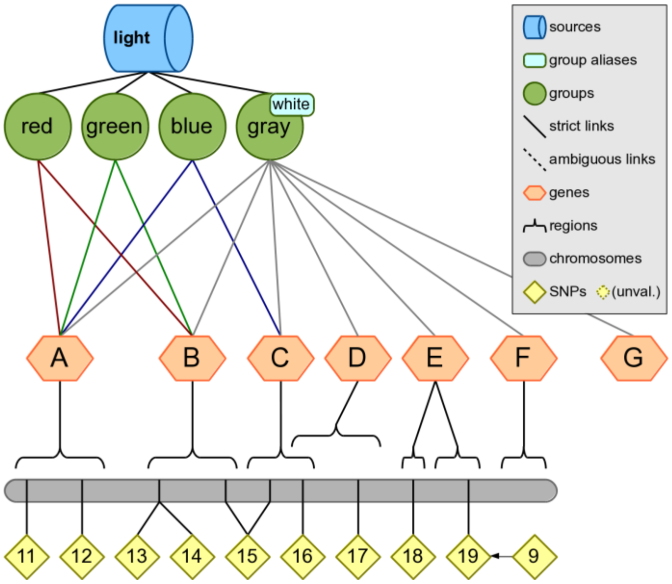

Example 5: Start with a list of genes, output all the genes within particular groups.

Input files:

input1	input2
#group<br>red<br>green<br>cyan<br>magenta<br>orange<br>indigo	#gene<br>A<br>C<br>E<br>P<br>R

Configuration:

```
KNOWLEDGE test.db
GROUP_FILE input1
GENE_FILE input2
FILTER region
```

Output:

```
#chr   region   start   stop
1      A        8       22
1      C        54      62
3      P        14      18
3      R        44      52
```

Example 6: Start with genes associated with a pathway or group, output genes within that group that overlap with an input list of genes.

Configuration:

```
KNOWLEDGE test.db
GENE P Q R
FILTER gene snp region group source
```

Output:

#gene	snp	chr	region	start	stop	group	source
Q	rs33	3	Q	28	36	orange	spectrum
Q	rs33	3	Q	28	36	indigo	spectrum
R	rs35	3	R	44	52	orange	spectrum
R	rs35	3	R	44	52	indigo	spectrum

Example 7: Starting with a list of genes, determine genes are within a group.

Configuration:

```
KNOWLEDGE test.db
GENE A C E H P Q R
GROUP cyan
FILTER gene group
```

Output:

#gene	group
A	cyan
C	cyan

Annotation Examples

Example 1: Annotating a SNP with gene region information.

Configuration:

```
KNOWLEDGE test.db
SNP rs11 rs24 rs99
ANNOTATE snp region
```

Output:

```
#snp   chr   region   start   stop
rs11   1     A        8       22
rs24   2     H        22      42
rs24   2     I        38      48
rs99
```

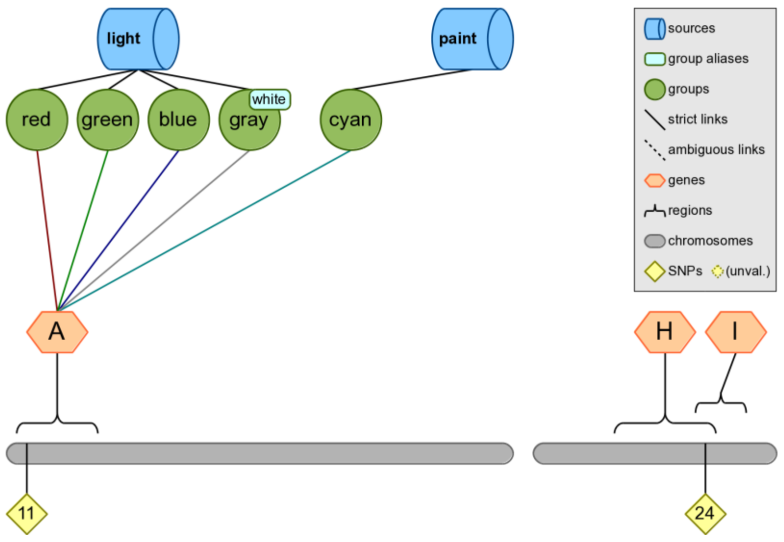

Example 2: Annotating SNPs with location information.

A user can provide Biofilter with a list of SNPs as an input and map those SNPs to the corresponding chromosome and base pair location (if any) as shown in the example below.

Configuration:

```
KNOWLEDGE test.db
SNP rs11 rs24 rs99
ANNOTATE snp position
```

Output:

```
#snp   chr   position   pos
rs11   1     rs11       10
rs24   2     rs24       40
rs99
```

Example 3: Map a SNP to the groups and sources where the SNP is present.

Biofilter can be used to map a list of SNPs, or a single SNP, to the groups and sources where those SNPs are present.

Configuration:

```
KNOWLEDGE test.db
SNP rs11 rs24 rs99
ANNOTATE snp group source
```

Output:

```
#snp   group   source
rs11   red     light
rs11   green   light
rs11   blue    light
rs11   gray    light
rs11   cyan    paint
rs24
rs99
```

Example 4: Annotating a base pair region with the list of SNPs in that region.

A region can be supplied to Biofilter, with an output of the SNPs known to be in that region.

Configuration:

```
KNOWLEDGE test.db
REGION 1:1:60
ANNOTATE snp region
```

Output:

```
#snp   chr   region   start   stop
rs11   1     A        8       22
rs12   1     A        8       22
rs13   1     B        28      52
rs14   1     B        28      52
rs15   1     B        28      52
rs15   1     C        52      62
rs16   1     C        54      62
rs16   1     D        58      72
```

Example Filtering followed by annotation

Example 1: Input a SNP list and map SNP positions to regions.

Configuration:

```
KNOWLEDGE test.db
SNP rs11 rs12 rs13 rs14 rs15 rs16
FILTER region
```

Output:

```
#chr    region    start    stop
1       A         8        22
1       B         28       52
1       C         54       62
1       D         58       72
```

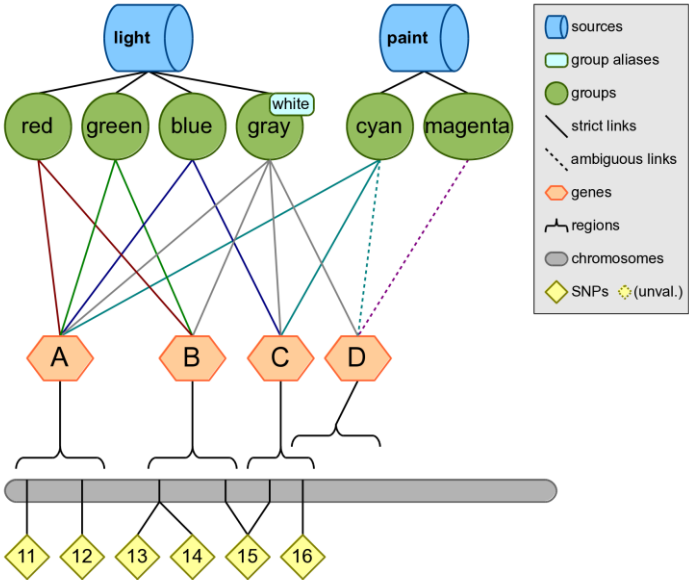

Example 2: Map SNPs to groups and filter on the source.

Configuration:

```
KNOWLEDGE test.db
SOURCE paint
FILTER snp group source
```

Output:

#snp	group	source
rs11	cyan	paint
rs12	cyan	paint
rs15	cyan	paint
rs16	cyan	paint

Example 3: Testing overlap of SNP and region lists, outputting regions.

Input files:

**input1**	**input2**			
#snp	#chr	region	start	stop
rs14	1	A	8	22
rs15	1	B	28	52
rs16	1	C	54	62
rs17	1	D	58	72
rs18				
rs19				

Configuration:

```
KNOWLEDGE test.db
SNP_FILE input1
REGION_FILE input2
FILTER region
```

Output:

#chr	region	start	stop
1	B	28	52
1	C	54	62
1	D	58	72

Example 4: Testing overlap of gene and source lists, outputting regions.

Configuration:

```
KNOWLEDGE test.db
GENE A C E G P R
SOURCE spectrum
FILTER region
```

Output:

```
#chr   region   start   stop
3      P        14      18
3      R        44      52
```

Example 5: Filter gene list based on sources, and output regions.

Configuration:

```
KNOWLEDGE test.db
GENE A C E G P R
SOURCE paint spectrum
FILTER gene source region
```

Output:

```
#gene   source     chr   region   start   stop
A       paint      1     A        8       22
C       paint      1     C        54      62
P       spectrum   3     P        14      18
R       spectrum   3     R        44      52
```

Example 6: Output of genes found in pathway based input, filtered by genotyping platform.

Configuration:

```
KNOWLEDGE test.db
SNP rs11 rs12 rs13 rs14
GENE A C E G P R
GROUP cyan yellow
FILTER region
```

Output:

```
#chr   region   start   stop
1      A        8       22
```

Example 7: Output of genes annotated by group found in pathway based input, filtered by genotyping platform.

Configuration:

```
KNOWLEDGE test.db
SNP rs11 rs12 rs13 rs14 rs16 rs17 rs18
GENE A C E G P R
GROUP cyan yellow
FILTER gene group
```

Output:

```
#gene   group
A       cyan
C       cyan
```

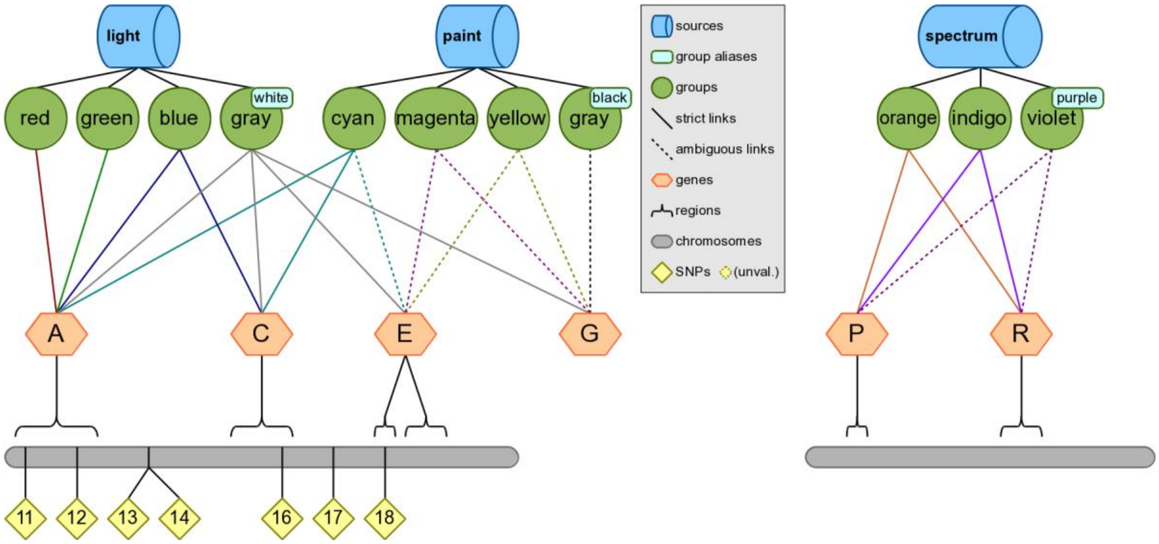

Example 8: Genes within data sources from a list of input genes filtered by genotyping platform, output regions.

Configuration:

```
KNOWLEDGE test.db
SNP rs11 rs12 rs13 rs14 rs16 rs17 rs18
GENE A C E G P R
SOURCE paint spectrum
FILTER region
```

Output:

#chr	region	start	stop
1	A	8	22
1	C	54	62

Example 9: Find overlap between two SNP lists and map the overlapping SNPs to the genes.

Input files:

**input1**	**input2**
#snp	#snp
rs11	rs14
rs12	rs15
rs13	rs16
rs14	rs17
rs15	rs18
rs16	rs19

Configuration:

```
KNOWLEDGE test.db
SNP_FILE input1
SNP_FILE input2
FILTER snp gene region
```

Output:

#snp	gene	chr	region	start	stop
rs14	B	1	B	28	52
rs15	B	1	B	28	52
rs15	C	1	C	54	62
rs16	C	1	C	54	62
rs16	D	1	D	58	72

Example 10: Find overlapping SNPs between the two lists and map the overlapping SNPs to the genes, regions, groups and the sources.

Configuration:

```
KNOWLEDGE test.db
SNP rs11 rs12 rs13 rs14 rs15 rs16
SNP rs14 rs15 rs16 rs17 rs18 rs19
FILTER snp gene region group source
```

Output:

#snp	gene	chr	region	start	stop	group	source
rs14	B	1	B	28	52	red	light
rs14	B	1	B	28	52	green	light
rs14	B	1	B	28	52	gray	light
rs15	B	1	B	28	52	red	light
rs15	B	1	B	28	52	green	light
rs15	B	1	B	28	52	gray	light
rs15	C	1	C	54	62	blue	light
rs15	C	1	C	54	62	gray	light
rs15	C	1	C	54	62	cyan	paint
rs16	C	1	C	54	62	blue	light
rs16	C	1	C	54	62	gray	light
rs16	C	1	C	54	62	cyan	paint
rs16	D	1	D	58	72	gray	light

Example 11: Mapping regions to genes using Biofilter based on percent of overlap.

Regions such as copy number variations can be mapped to genes using Biofilter, carried out based on percent of overlap of the genes with the CNV region or based on the number of base pairs overlapped.

For reference, here are the boundary positions for the genes in chromosome 1:

#chr	gene	start	stop
1	A	8	22
1	B	28	54
1	C	54	62
1	D	58	72
1	E	78	82
1	E	84	92
1	F	94	98

Configuration:

```
KNOWLEDGE test.db
REGION 1:1:60
REGION_MATCH_PERCENT 50
FILTER gene
```

Output:

```
#gene
A
B
C
```

This output indicates that at least 50% of genes A, B, and C fall within the first 60 bases of the first chromosome. Both genes A and B match 100% of the region while gene C matches 75%.

Example 12: Mapping regions to genes using Biofilter based on base pair overlap.

The genes overlapping region based on number of base-pair overlap can also be determined via Biofilter.

Configuration:

```
KNOWLEDGE test.db
REGION 1:1:60
REGION_MATCH_BASES 10
FILTER gene
```

Output:

```
#gene
A
B
```

This output uses the region-match-bases argument to specify that Biofilter should filter genes that only match a minimum of 10 bases within the given input region.

Example 13: Annotating a list of gene symbols with SNPs, regions, groups, and sources, using Biofilter.

Configuration:

```
KNOWLEDGE test.db
GENE A B C D E
FILTER gene snp region group source
```

Output:

```
#gene   snp    chr   region   start   stop   group   source
A       rs11   1     A        8       22     red     light
A       rs11   1     A        8       22     green   light
A       rs11   1     A        8       22     blue    light
A       rs11   1     A        8       22     gray    light
A       rs11   1     A        8       22     cyan    paint
A       rs12   1     A        8       22     red     light
A       rs12   1     A        8       22     green   light
A       rs12   1     A        8       22     blue    light
A       rs12   1     A        8       22     gray    light
A       rs12   1     A        8       22     cyan    paint
B       rs13   1     B        28      52     red     light
B       rs13   1     B        28      52     green   light
B       rs13   1     B        28      52     gray    light
```

B	rs14	1	B	28	52	red	light
B	rs14	1	B	28	52	green	light
B	rs14	1	B	28	52	gray	light
B	rs15	1	B	28	52	red	light
B	rs15	1	B	28	52	green	light
B	rs15	1	B	28	52	gray	light
C	rs15	1	C	54	62	blue	light
C	rs15	1	C	54	62	gray	light
C	rs15	1	C	54	62	cyan	paint
C	rs16	1	C	54	62	blue	light
C	rs16	1	C	54	62	gray	light
C	rs16	1	C	54	62	cyan	paint
D	rs16	1	D	58	72	gray	light
D	rs17	1	D	58	72	gray	light
E	rs18	1	E	78	82	gray	light
E	rs19	1	E	84	92	gray	light

Modeling Example

Here we present an example with two sources and eight pathways shown in the below figure, to explain how Biofilter can generate pairwise SNP-SNP and Gene-Gene models. In further sections we explain other options for how model generation can be performed in Biofilter 2.0.

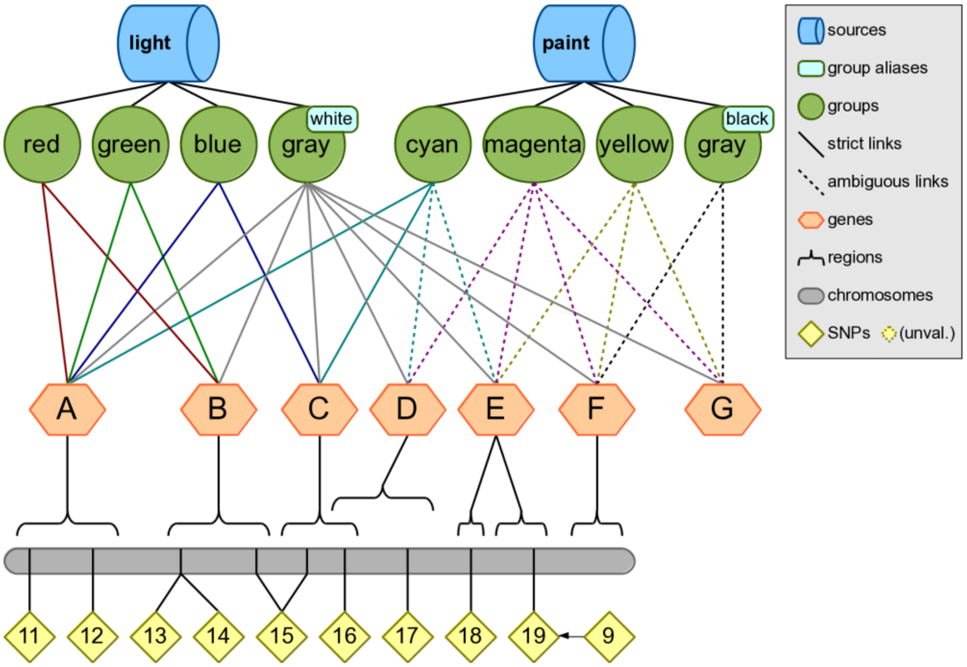

Step 1

Map the input list of SNPs to genes within Biofilter; for this example, we will use all of the SNPs on the first chromosome. Note that Gene F does not contain any SNPs.

Configuration:

```
KNOWLEDGE test.db
SNP 11 12 13 14 15 16 17 18 19
FILTER gene
```

Output:

```
#gene
A
B
C
D
E
```

Step 2

Connect, pairwise, the genes that contain SNPs in the input list of SNPs.

Configuration:

```
KNOWLEDGE test.db
GENE A B C D E
MODEL gene
```

Output:

```
#gene1   gene2   score(src-grp)
A        C       2-3
```

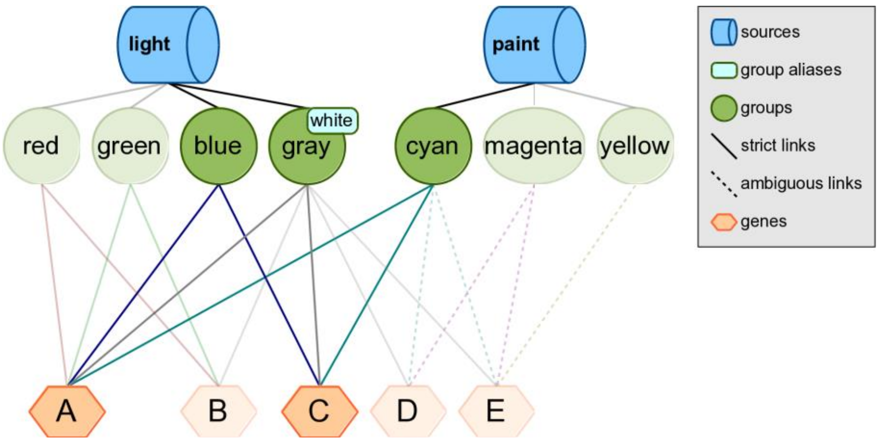

Biofilter has determined that genes A and C are found together in three groups across two sources. In other words, both the *light* and *paint* sources contain groups—blue, gray, and cyan—that suggest a relationship between genes A and C.

This relationship is summarized by the implication score "2-3," which gives the number of sources followed by the number of groups which support this gene model. Each time the same pairwise model of genes is found in another source, the left-hand index of the implication score for that pairwise model increases by one; each time it is found in another group from the same source, the right-hand index increases by one.

Step 3

Break down the gene-gene models into all pairwise combinations of SNPs across the genes within sources *light* and *paint*.

Configuration:

```
KNOWLEDGE test.db
SOURCE light paint
MODEL snp
```

Output:

```
#snp1   snp2   score(src-grp)
rs11    rs15   2-3
rs11    rs16   2-3
rs12    rs15   2-3
rs12    rs16   2-3
```

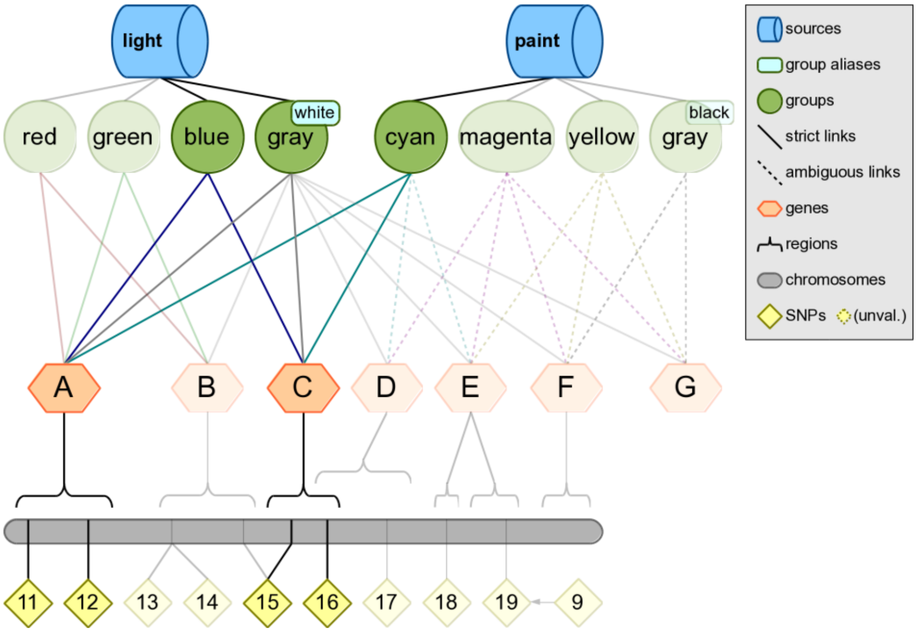

Changes in Biofilter 2.0 Modeling

Although this three-step strategy will work in the new version of Biofilter, the strategy can be simplified. Biofilter 2.0 will automatically generate gene models prior to generating SNP models and there is no need to specify that step separately. It is possible to generate the SNP models with a single command.

Configuration:

```
KNOWLEDGE test.db
SNP 11 12 13 14 15 16 17 18 19
MODEL snp
```

Output:

```
#snp1   snp2   score(src-grp)
rs11    rs15   2-3
rs11    rs16   2-3
rs12    rs15   2-3
rs12    rs16   2-3
```

Appendix 1: Ambiguity in Prior Knowledge

When an ambiguous gene or group identifier appears in a user input file, Biofilter has two straightforward options: either include all genes or groups with which the identifier is associated, or none of them.

When processing the bulk downloads from prior knowledge sources, however, the situation can become more complicated. This is due to the fact that in many cases, the data provided by a source is formatted in a way which allows multiple identifiers to be provided for the same member of a group. Ideally all such identifiers are known to refer to the same single gene, but occasionally this is not the case. Sometimes one of the identifiers is an alias of more than one gene, making it inherently ambiguous; other times, even if every identifier refers to only one gene, they might not all agree on which gene that is.

The testing knowledge included with Biofilter contains several examples of these kinds of situations, depicted in the diagram below. Note that this diagram reflects the fact that there may be multiple names for the same gene (i.e. "D" and "DE" both refer to gene D), and some names may be associated with multiple genes (i.e. "DE" refers to both genes D and E).

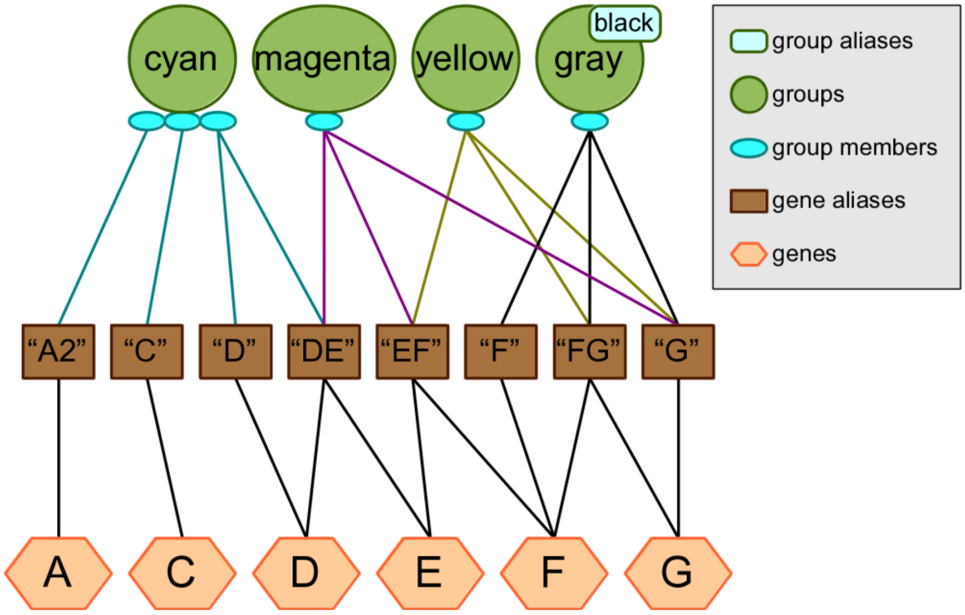

The "cyan" group contains three genes, of which the third is ambiguous because we are given two identifiers for it, but one of them refers to two different genes. The "magenta", "yellow" and "gray/black" groups each contain only one gene, but in each case we are given three different names for that gene which agree or disagree with each other in varying ways. Because of the ambiguity in the provided identifiers, the genes which are considered members of these groups will appear to vary depending on the user's choice for the ALLOW_AMBIGUOUS_KNOWLEDGE and REDUCE_AMBIGUOUS_KNOWLEDGE options.

Ambiguity Reduction Heuristics

Biofilter and LOKI currently support two heuristic strategies for reducing ambiguity. These strategies make what is essentially an educated guess about what the original data source intended by the set of identifiers it provided. The first heuristic is called "implication" and it rates the likelihood of each potential gene being the intended one by counting the number of identifiers which implicate that gene. The second heuristic, called "quality," is similar except that it also considers the number of genes that each identifier refers to as a measure of that identifier's quality; a high-quality identifier (which refers to only one or two genes) is then given more weight than a low-quality identifier (which refers to many genes).

In practice, these two heuristic strategies will often produce the same results; in fact, when using real data from our real prior knowledge sources, we have yet to find a case where they do not reach the same conclusion. It is possible that such a case will arise in the future, however, so the "magenta", "yellow" and "gray/black" groups in the testing knowledge have been specially crafted to highlight these potential differences.

Ambiguity Options

The REDUCE_AMBIGUOUS_KNOWLEDGE option tells Biofilter which heuristics, if any, should be employed to mitigate ambiguity in the prior knowledge database. The permissible values for this option are the name of any of the heuristic strategies, or "no" or "any". When set to "no" then no attempt is made to reduce ambiguity and all genes which are implicated by any of the provided identifiers are considered equally likely interpretations. When set to "any" then all heuristics are attempted simultaneously and the winner(s) from each one collectively become the preferred choices.

The ALLOW_AMBIGUOUS_KNOWLEDGE option tells Biofilter what to do when it has more than one "best guess" interpretation for an ambiguous member of a group. If no heuristics were used then this occurs for all cases of ambiguity, but it should also be noted that any heuristic strategy might be only partly successful. For example, if a given set of identifiers collectively refer to three different genes and the heuristic(s) can only eliminate one of them, then the other two remain equally likely possibilities. In cases like this, the user's choice for ALLOW_AMBIGUOUS_KNOWLEDGE determines the result: when disabled (the strict option) none of the possible genes will be considered a member of the group, but when enabled (the permissive option) the most-likely possibilities will all be included, without any of the less-likely possibilities.

Gene Ambiguity Examples

Example 1: cyan

The "cyan" group is a typical case of ambiguity which can be fully resolved by either of the heuristic strategies. Its first two members (genes A and C) are unambiguous and will always be included, but the correct third member of the group is open to interpretation.

The implication heuristic will declare D as the correct interpretation since it is implicated by both of the provided identifiers, while gene E is only implicated by one of them.

The quality heuristic will also choose D, but its reasoning is a little more involved. The "DE" identifier refers to two different genes, so it gets a quality score of 1/2 or 0.5; the "D" identifier, on the other hand, gets a quality score of 1 because it refers to only one gene. Gene E therefore receives only 0.5 points, while gene D wins with 1.5 total points.

Because the ambiguity can be fully resolved by either heuristic, the ALLOW_AMBIGUOUS_KNOWLEDGE option will only have an effect if no heuristics are used at all. In that case, the group will contain all four possible genes (A, C, D and E) if the option is enabled, but only A and C if it is disabled.

Example 2: magenta

The "magenta" group demonstrates ambiguity which can only be resolved by the implication heuristic: gene E is implicated by two identifiers ("DE" and "EF") while genes D, F and G are each only implicated by one identifier.

The quality heuristic will discard genes D and F (0.5 points each), but cannot pick a winner between E and G because they both have a score of 1.0: gene E gets 0.5 each from the "DE" and "EF" identifiers, while gene G gets 1 full point from "G".

With no heuristics, the ALLOW_AMBIGUOUS_KNOWLEDGE option will either include all four genes or none of them. With the quality heuristic, it can either include both winners (E and G) or nothing. With the implication heuristic it has no effect here, since the ambiguity was eliminated with that strategy.

Example 3: yellow

The "yellow" group demonstrates ambiguity which can only be resolved by the quality heuristic: gene G wins with a score of 1.5 (0.5 from "FG" plus 1.0 from "G").

The implication heuristic will discard gene E (implicated by only one identifier), but cannot choose between F and G because they are implicated by two identifiers each: "EF and "FG", or "FG and "G".

With no heuristics, as always ALLOW_AMBIGUOUS_KNOWLEDGE will either include every possibility or none of them. With the implication heuristic it can either include both F and G or nothing, and with the quality heuristic it has no effect.

Example 4: gray/black

The "gray/black" group is an example of ambiguity which cannot be resolved by either heuristic: genes F and G are entirely comparable, both being referenced by one specific identifier plus one (shared) ambiguous identifier. No matter which heuristic is used, if any, this group will always contain both F and G if ALLOW_AMBIGUOUS_KNOWLEDGE is enabled, or neither if it is disabled.

Protein Identifiers

So far, our depiction of ambiguity in the knowledge database has implied that groups always contain genes. This allows for the convenient assumption that when we are given more than one identifier for something in a group, we are expecting all of those identifiers to refer to one (and only one) gene.

The reality is, of course, a little more complicated: some sources provide groups which actually contain proteins. In order to make this knowledge compatible with the rest of the prior knowledge, LOKI must translate these protein references into genes, but this breaks that convenient assumption. If a group contains genes then we can reasonably expect each member of the group to be a single gene, but if the group contains proteins, then we must be prepared for a single protein-member to correspond to many genes.

To account for this, LOKI differentiates between identifiers which refer directly to genes (such as symbolic abbreviations or Entrez Gene ID numbers) and identifiers which refer to proteins (such as UniProt ID numbers) that may in turn correspond to many genes.

If any of the identifiers provided for one member of a group is a protein identifier, LOKI disregards any non-protein identifiers. If there is only one protein identifier, then LOKI considers all genes which correspond to that protein to be members of the group, with no ambiguity. If there are multiple protein identifiers then there may be ambiguity if they do not correspond to the same set of genes.

Since protein identifiers are expected to correspond to multiple genes, the concept of an identifier's "quality" no longer has meaning; consequently, whenever protein identifiers are involved, the implication and quality heuristic strategies become functionally equivalent. In both cases, a gene's likelihood of being associated with a group is proportional to the number of protein identifiers which implicated it. When no heuristics are used, then all genes which are implicated by any of the protein identifiers are considered equally likely to belong in the group.

The testing knowledge included with Biofilter also contains several examples of groups with protein identifiers:

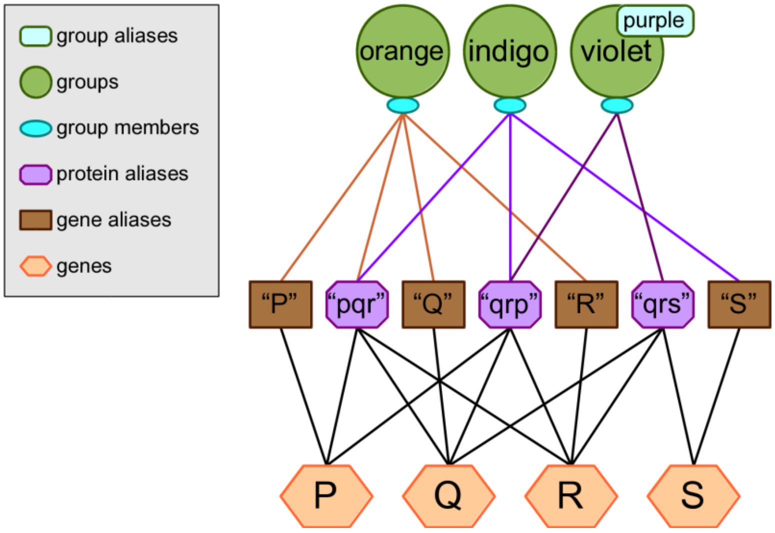

Protein Ambiguity Examples

Example 1: orange

The “orange” group contains a simple, unambiguous use of protein identifiers. No matter what options are used, this group will always contain the genes P, Q and R.

Example 2: indigo

The “indigo” group demonstrates a more complicated but still unambiguous situation. The two protein identifiers agree with each other, so the group will always contain the genes P, Q and R no matter what options are used. However there is an extraneous gene identifier which is ignored, even though it does not appear to match the protein identifiers. In practice, this is rarely the case; when a source provides both protein and gene identifiers, the latter usually agree with the former.

Example 3: violet

In the “violet” group the two protein identifiers only partly agree: both of them correspond to genes Q and R, but one of them also matches P while the other also matches S.

If ALLOW_AMBIGUOUS_KNOWLEDGE is enabled then all four genes will be included in the group. If it is disabled, then any heuristic strategy will include genes Q and R but not P or S. If no heuristics are used either, then the group will appear empty.

Appendix 2: LD Profiles

Each LD profile is defined by two things: a reference population whose particular LD patterns are relevant to the user's analysis, and a threshold value to specify what the user considers "high LD." Every LOKI knowledge database file begins with a default, unnamed LD profile which contains the canonical gene region boundaries and therefore has no reference population or LD threshold. All other LD profiles are calculated based on these original boundaries, which means whenever the LOKI knowledge database is updated, the LD profiles must also be re-calculated to incorporate any changes in the original gene region boundaries.

In order to generate LD profiles, Biofilter is distributed with a separate software tool called LD Spline. This tool can use specific LD measurements from the HapMap project to extrapolate more general LD patterns, which can in turn be used to calculate LD profiles containing adjusted gene region boundaries. More information about LD Spline is available from www.ritchielab.psu.edu.

Installing LD Spline

LD Spline is written in C and must therefore be compiled for your local computing environment before it can be used. To do this automatically as part of the Biofilter installation process, simply use the "--ldprofile" option:

```
python setup.py install --ldprofile
```

This will compile and install the "ldspline" executable, along with a few supporting scripts which automate the process of generating and storing LD profiles in LOKI.

Generating LD Profiles

The LOKI prior knowledge database file must be generated before the LD adjustment can be done; refer to the Biofilter installation instructions for details on this procedure. Once the knowledge file is available, use the "buildPopulations.py" script to generate additional LD profiles. For example:

```
buildPopulations.py --db loki.db --populations CEU,YRI --dprime 0.6,0.7
                   --rsquared 0.8,0.9
```

This will generate 8 additional LD profiles for use in LOKI and Biofilter: four each for the CEU and YRI populations, of which two represent the LD pattern using D' thresholds of 0.6 and 0.7 and the other two use the R^2 metric with thresholds of 0.8 and 0.9. Note that building LD profiles may take quite some time; plan for at least 2 hours per population when run on a local disk, or twice that on a networked filesystem (such as GPFS). The build process also requires ~2 GB of RAM and some temporary disk space in the working directory: allow for 10 GB, plus another 10 GB per population.

With the modified knowledge database file, Biofilter can then make use of the alternate gene regions via the LD_PROFILE option:

```
biofilter.py --knowledge loki.db --ld-profile CEU-RS0.80
```

The "--report-ld-profiles" option can be used to list the LD profiles available in a LOKI database file:

```
biofilter.py --knowledge withld.db --report-ld-profiles
```

```
#ldprofile  description                                         metric     value
            no LD adjustment
CEU-DP0.60  CEU population from HapMap with dprime cutoff 0.6      dprime     0.6
CEU-DP0.70  CEU population from HapMap with dprime cutoff 0.7      dprime     0.7
CEU-RS0.80  CEU population from HapMap with rsquared cutoff 0.8    rsquared   0.8
CEU-RS0.90  CEU population from HapMap with rsquared cutoff 0.9    rsquared   0.9
YRI-DP0.60  YRI population from HapMap with dprime cutoff 0.6      dprime     0.6
YRI-DP0.70  YRI population from HapMap with dprime cutoff 0.7      dprime     0.7
YRI-RS0.80  YRI population from HapMap with rsquared cutoff 0.8    rsquared   0.8
YRI-RS0.90  YRI population from HapMap with rsquared cutoff 0.9    rsquared   0.9
```

Population Build Script Options

--help
Displays the program usage and immediately exits.

--populations
A comma-separated list of 3-letter HapMap population identifiers (i.e. "CEU", "JPT", "YRI", etc.)

--rsquared
A comma-separated list of R^2 threshold values (between 0 and 1) for which to generate LD profiles.

--dprime
A comma-separated list of D' threshold values (between 0 and 1) for which to generate LD profiles.

--liftover
The location of UCSC's liftOver utility, which is needed to convert HapMap's LD measurements to the current reference genome build. If omitted, liftOver must be available on the path.

--ldspline
The location of the LD Spline utility, which will be installed by the Biofilter installer if given the "--ldprofile" option. If omitted, ldspline must be available on the path.

--poploader
The location of the pop_loader helper script, which will be installed by the Biofilter installer if given the "--ldprofile" option. If omitted, pop_loader must be available on the path.

--db
The LOKI prior knowledge database file in which to generate LD-adjusted gene regions. The database must already contain the canonical gene regions.

--keep-data
Generating LD profiles requires many intermediate files such as original LD data from HapMap and extrapolated LD data from LD Spline. By default these intermediate files are deleted after use; if this option is specified, they will be left in place.
